# Real-world mileage and survival of electric vehicles in Britain and implications for lifecycle emissions

**DOI:** 10.1038/s41467-026-77892-4

**Published:** 2026-09-21

**Authors:** Daniel Mehlig, Iain Staffell

**Affiliations:** Centre for Environmental Policy, Imperial College London, London, UK

**Keywords:** Energy science and technology, Civil engineering, Computer science

## Abstract

Battery electric vehicles (BEVs) are typically estimated to have lower attributional lifecycle greenhouse gas emissions than conventional internal combustion engine vehicles. These estimates rely upon lower usage emissions outweighing higher manufacturing emissions, but limited data on electric vehicle mileage, and how long they last, casts uncertainty over comparisons. Here we use data for every electric vehicle in Britain to assess mileage and lifetime compared to conventional and hybrid vehicles, and the implications for attributional lifecycle emissions. We find that first-generation BEVs were driven less and have not lasted as long as conventional vehicles, yet still had lower lifecycle emissions. The annual mileage and lifetime of BEVs purchased in 2026 will equal conventional vehicles if recent trends continue. Training a transformer neural network on this data, we estimate that current BEVs have projected attributional lifecycle emissions of 88 g CO_2e_ per kilometre, less than half the 254-270 g for conventional vehicles and 190 g for hybrid vehicles, based on real-world usage.

## Abstract (web summary)

*Analysis of Britain’s entire vehicle fleet finds electric cars now match petrol and diesel cars in mileage and lifespan and produce less than half the lifetime greenhouse gas emissions of conventional vehicles, based on real-world usage data*.

## Introduction

**Fig. 1:**
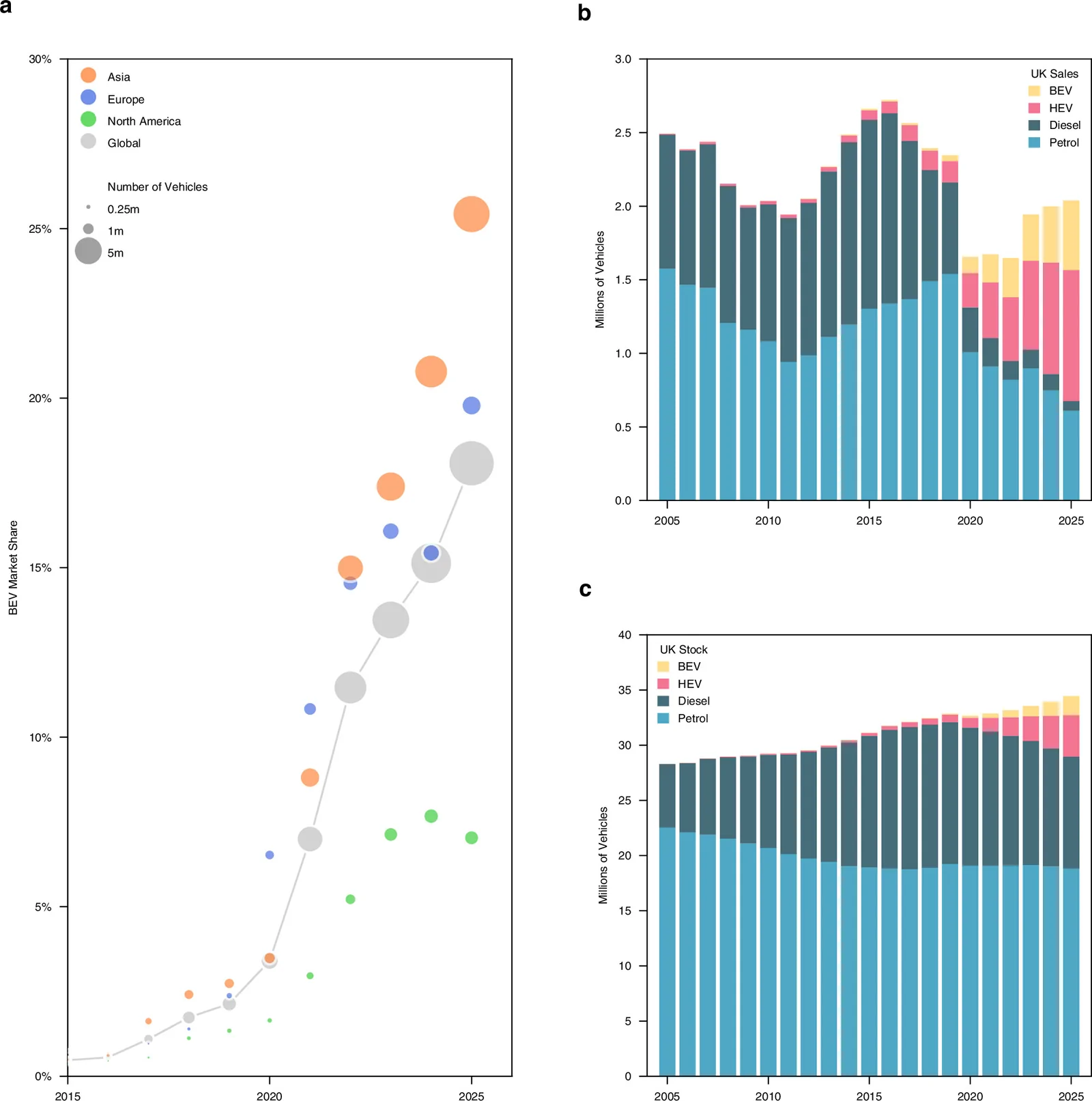
Electric vehicle sales and fleet size. **a** gives global battery electric vehicle car sales as a proportion of the market where the size of the marker indicates the number of sales for the market and the colour shows the market using data compiled by Andrew^76^; (**b**) provides UK car sales by fuel type, where hybrid electric vehicles include Plug-ins; (**c**) provides the overall UK car stock using the same colour scheme as (**b**),(**b**, **c**) use values from the UK’s Department of Transport’s online licensing statistics^25^. Abbreviations: Battery Electric Vehicle (BEV), Hybrid Electric Vehicle (HEV).

Electric Vehicles (EVs) are becoming mainstream. This follows growing government and consumer interest over the past decade, resulting in the global market share approaching one fifth of all newly registered vehicles^1^ as shown in Fig. 1a. A primary motivation for electrifying transport is the potential to lower greenhouse gas emissions over their lifetime compared to incumbent combustion-engine drivetrains^2–5^. Attributional lifecycle emissions comparisons rely on four factors: embodied manufacturing emissions, use-phase emissions from electricity generation supplying the vehicles, how much vehicles are driven, and how long vehicles last. The first two factors have been widely studied^2–5^ showing that EV manufacturing emissions are higher than existing vehicle technologies, but these are expected to be outweighed by lower use-phase emissions when vehicle mileage and lifetime are equal^2–5^. So far, this evidence has been prospective, illustrating the theoretical potential of these vehicles rather than their real-world emissions footprint. The use phase – understanding how vehicles will be used and how long they will last in the fleet – has so far only been estimated, with existing work typically assuming the same usage patterns and lifetimes as conventional vehicle technologies^3–7^.

Initial evidence suggests that EVs have not yet been used with the same patterns as conventional vehicles^8–12^. The first generation of EVs had limited battery sizes and range^13^ alongside sparse charging infrastructure^14^, leading to altered driving behaviour known as “range anxiety”^15^. In addition, early EVs with smaller battery capacities may have reached end-of-life degradation thresholds sooner, as cycling rates were higher compared to newer models with larger battery packs. As EVs and their supporting infrastructure improve, these early constraints on durability and usage are likely changing. How this translates into real-world vehicle lifecycle emissions remains poorly evidenced, due to the scarcity of robust empirical data on how EV mileage and lifetime are evolving.

Here we use data giving the usage and lifetime of all vehicles in Britain, including Battery Electric Vehicles (BEVs) and Hybrid Electric Vehicles (HEVs) over the twenty years from 2005 to 2025. We assess how all BEVs and HEVs have been driven and how long they have lasted in the fleet so far, comparing these to a representative sample of petrol and diesel vehicles. We combine data on vehicle mileage and lifetime with vehicle attributes, such as fuel economy, battery size, and vehicle mass, to calculate attributional emissions of vehicles over their lifetime. The majority of Britain’s BEVs and HEVs are still on the road today and have not reached the end of their life. Therefore, we use a transformer neural network model, trained on this national dataset to project the lifetime and future driving patterns of existing vehicles in the fleet, to estimate attributional emissions over their lifetime.

Every car in Britain must pass a yearly roadworthiness test from its third year onwards, the Ministry of Transport (MOT) test. Results, including vehicle attributes and mileage, have been recorded, anonymised, and made publicly available by the UK government for tests from 2005 onwards^16^. This data has previously been used to derive how conventional vehicles are driven and scrapped as they age for air pollutant and greenhouse gas emissions analysis^9,10,17–19^ and for calculating other transport-related socio-economic metrics^10,20,21^. We cross-reference vehicle characteristics, such as the fuel consumption for ICEVs and the battery size and energy consumption for BEVs, into the MOT data using three vehicle databases^13,22,23^ (see Methods) following Newman’s approach^19^. Both mild and full hybrids are labelled as HEVs in UK statistics and vehicle databases^24^, whereas plug-in hybrids (PHEVs) were excluded from this study as their share of fuel and electricity consumption is not disaggregated in the available databases, meaning emissions estimates would rely upon generalised assumptions.

## Results

### Vehicle mileage

**Fig. 2:**
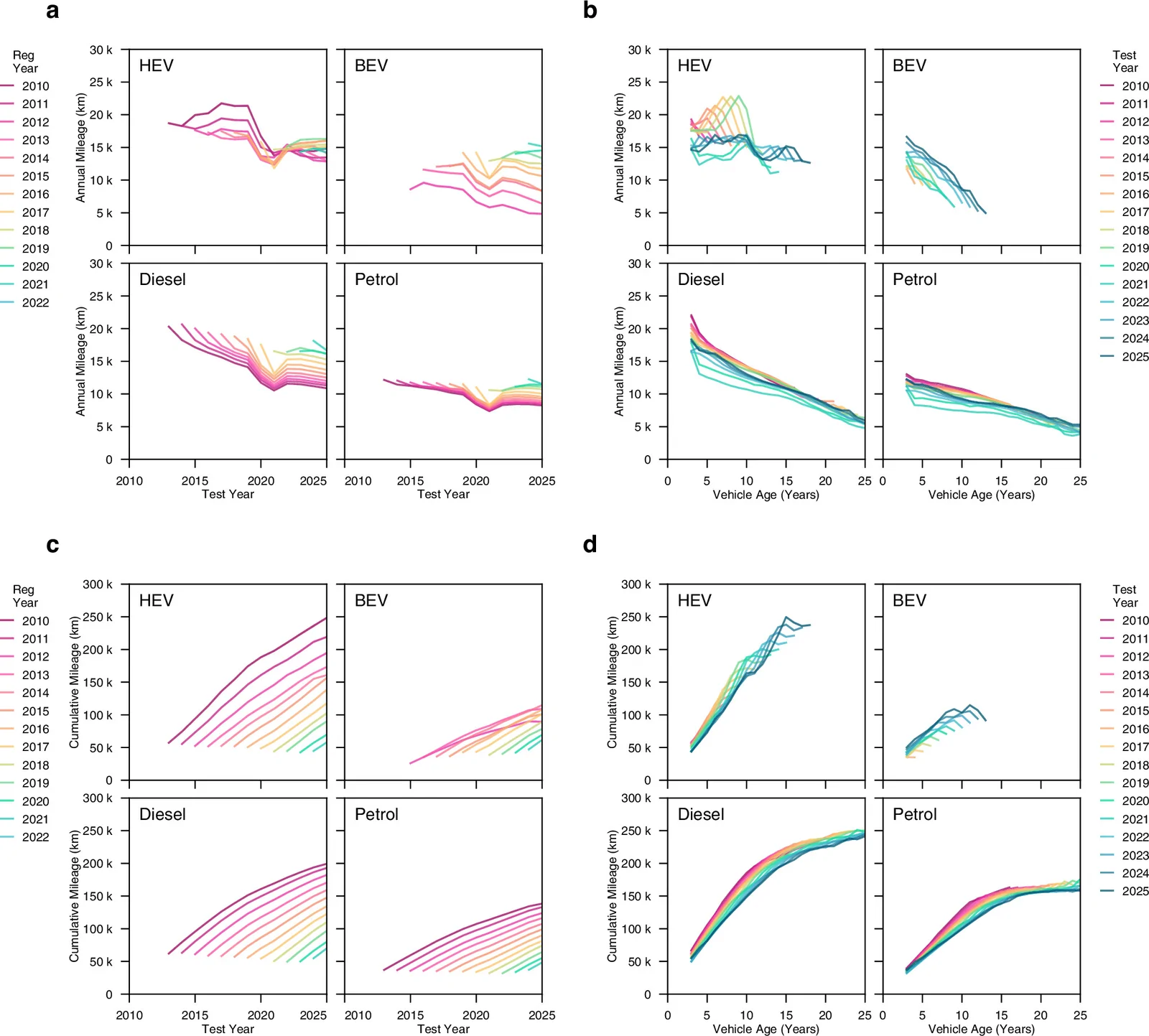
Distance driven by vehicles in the UK as a function of age and type. Annual (**a**, **b**) and cumulative (**c**, **d**) mileage for hybrid electric vehicles, battery electric vehicles, diesel, and petrol non-hybrid cars. **a** gives the mean annual mileage for each test year between 2013 and 2025 and is disaggregated by colour, with each line showing the mean across each vehicle registration year (each cohort of vehicles); **b** gives the mean annual mileage by vehicle age across all registration years, where each coloured line shows the test year. Panels (**c**, **d**) repeat (**a**, **b**), but give the mean cumulative mileage. All lines are given with accompanying area plots showing the standard error of the mean, where most areas are not visible since they fall underneath the central line. Abbreviations: Battery Electric Vehicle (BEV), Hybrid Electric Vehicle (HEV), Registration Year (Reg Year). Source data are provided, giving sample sizes and underlying data for the figure.

**Fig. 3:**
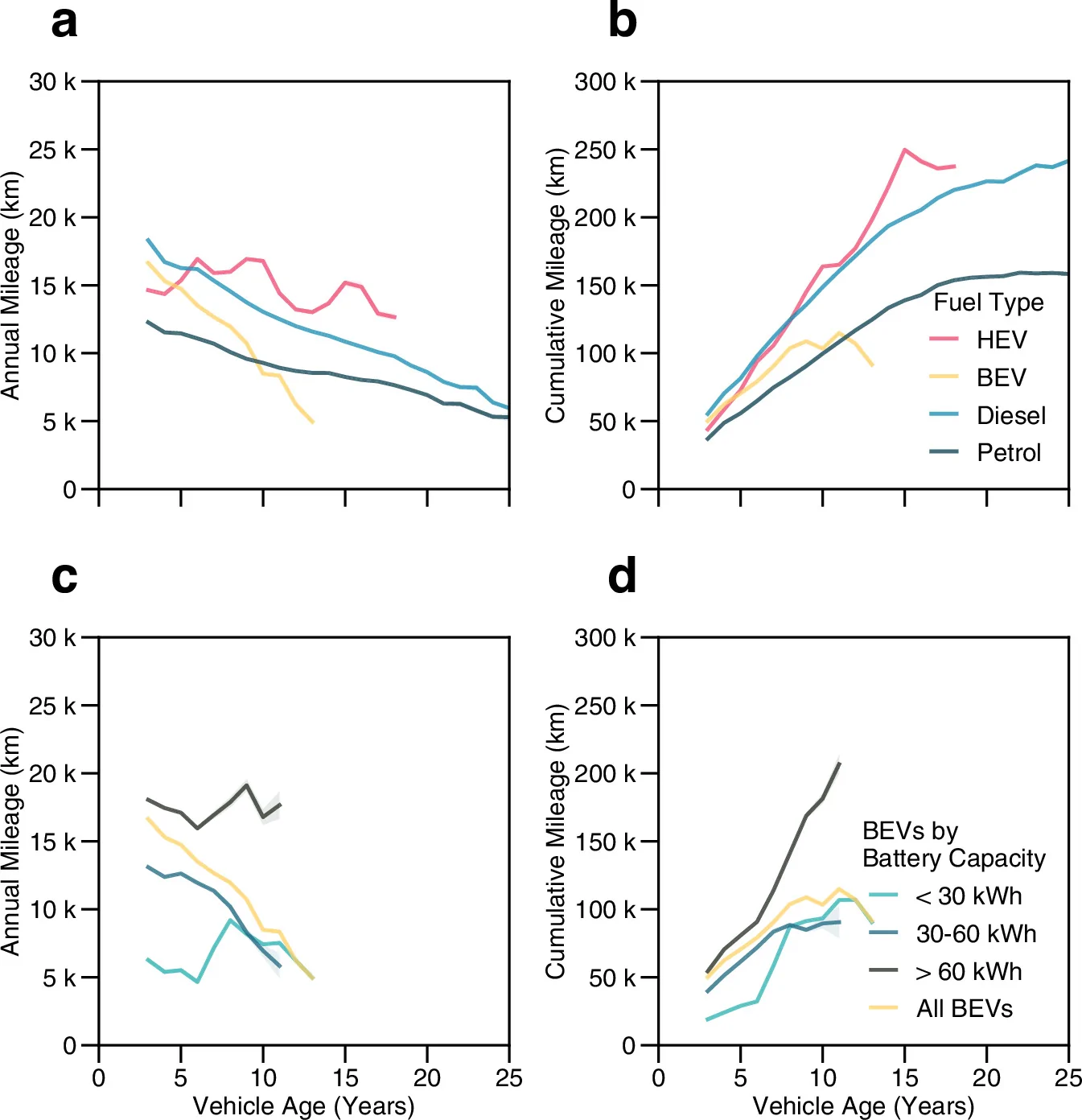
Distance driven by vehicles in the UK by fuel type and battery size. Annual (**a**, **c**) and cumulative (**b**, **d**) mileage for hybrid electric vehicles, battery electric vehicles, diesel, and petrol non-hybrid cars. **a** gives the mean annual mileage, and (**b**) gives the mean cumulative lifetime mileage, in 2025, where fuel type is shown by colour. Panels (**c**, **d**) repeat (**a**, **b**), but disaggregate BEVs by battery capacity. All lines are given with accompanying area plots showing the standard error of the mean, where most areas are not visible since they fall underneath the central line. Abbreviations: Battery Electric Vehicle (BEV), Hybrid Electric Vehicle (HEV). Source data are provided, giving sample sizes and underlying data for the figure.

The MOT data was used to calculate the annual and cumulative mileage for vehicles in the UK across time. Figure 2 and Fig. 3 show how the usage of each vehicle fuel type has evolved, and the impact of external factors such as reduced annual mileage during and since the Covid-19 pandemic (Fig. 2a). The annual and cumulative mileage for each registration year, starting from 2010 for diesel, petrol and HEVs, and starting from 2012 for BEVs are shown in Fig. 2a, c for each year of the MOT test data. These data were translated from registration years into fleet-wide profiles that give the overall relationship between mileage and vehicle age for each vehicle fuel type in Fig. 2b, d.

Figure 2 shows that diesel and petrol vehicles are being used less intensively than in the past. The annual mileage of new diesel vehicles (those aged three that enter the MOT data) has reduced from 22,050 km per year in 2010 to 18,280 km per year in 2025, while for petrol vehicles the fall is from 13,030 to 12,270 km per year. The annual mileage of diesel and petrol vehicles also declines as they age. The rate of decline is lower in more recent years, as older vehicles are being driven similar distances as they were in the past. Each year a diesel vehicle ages, it is driven a mean of 610 km per year less in 2025 with an associated ± 30 km per year standard error (down from 815 ± 50 km per year in 2010), while petrol vehicles are driven 340 ± 20 km per year less (unchanged from 330 ± 10 km per year in 2010), based on the trends in Fig. 2b over the first 15 years of operation. Put together, these trends have resulted in conventional vehicles aged ten having accrued 30,080–36,910 km lower cumulative mileage in 2025 than in 2010. This downward trend aligns with national shifts in the number of vehicles and kilometres driven nationally in the UK, where distance driven per vehicle has declined by 7% between 2010 and 2025^25,26^.

As HEVs and BEVs are newer vehicle classes, their usage has not yet reached a stable pattern, with trends each year towards lower annual mileage for HEVs and higher annual mileage for BEVs. Both technologies are converging towards a utilisation pattern between petrol and diesel vehicles, as shown in Fig. 2b and Fig. 3a. For new HEVs, annual mileage has declined from 17,860 km per year in 2015 to 14,620 km per year in 2025 and the decline in mileage with age in 2025 for HEVs was 130 ± 100 km per year when using the trends given in Fig. 2b over the first 15 years.

The annual mileage of new BEVs has increased from 8,480 km per year in 2015 to 16,650 km per year in 2025. Since 2017, the annual mileage of new BEVs has exceeded that of petrol vehicles. The decline in annual mileage with vehicle age was largest for BEVs compared to the other fuel types, at 1140 ± 50 km per year in 2025. This is confounded by cohort effects in the BEV fleet, as older vehicles with smaller battery capacities and shorter range are being driven less than newer vehicles, giving a steeper decline in annual mileage across the latest MOT test year.

We disaggregate the BEVs by battery capacity in Fig. 3c and Fig. 3d to show how evolving technology impacts annual mileage and cumulative mileage of BEVs. Larger BEVs ( > 60 kWh, such as the Tesla Model S or Jaguar I-Pace) had the highest annual mileage of 18,050 km per year when new, while smaller BEVs ( < 30 kWh, such as the original Nissan Leaf or Renault Zoe) were only driven 6270 km per year. This shows significant variability within the BEV fuel type, where models with the largest range and batteries are driven as intensively as HEVs and diesel vehicles.

### Vehicle survival

There is interest surrounding BEV lifetime since existing lifecycle emissions comparisons rely upon lifetimes being equivalent to conventional vehicle drivetrains^2–5^. We used the MOT data to calculate the survival rate, the proportion of vehicles that remain in the fleet as vehicles age. Survival rates were calculated using a cohort approach, recording how many vehicles entered the fleet for each registration year and tracking how many remain as the cohorts age. Vehicles have their first MOT test at age 3, so we combine this data with the Vehicle Licensing Statistics dataset^25^ to account for the proportion of vehicles that exit in these first three years. This allowed us to use the same cohort-based approach as has been previously used to assess survival rates^9,17,27^.

We use the criteria from Nguyen-Tien et al.^10^ to determine if a vehicle has exited the fleet which allows us estimate vehicle survival up until tests taken in 2023. The use of the Vehicle Licensing Statistics^25^ dataset to account for vehicle survival in the first three years has minimal impact for conventional vehicles, but makes an important correction to the survival rates of BEVs published in previous work^10^, especially for pre-2017 models.

**Fig. 4:**
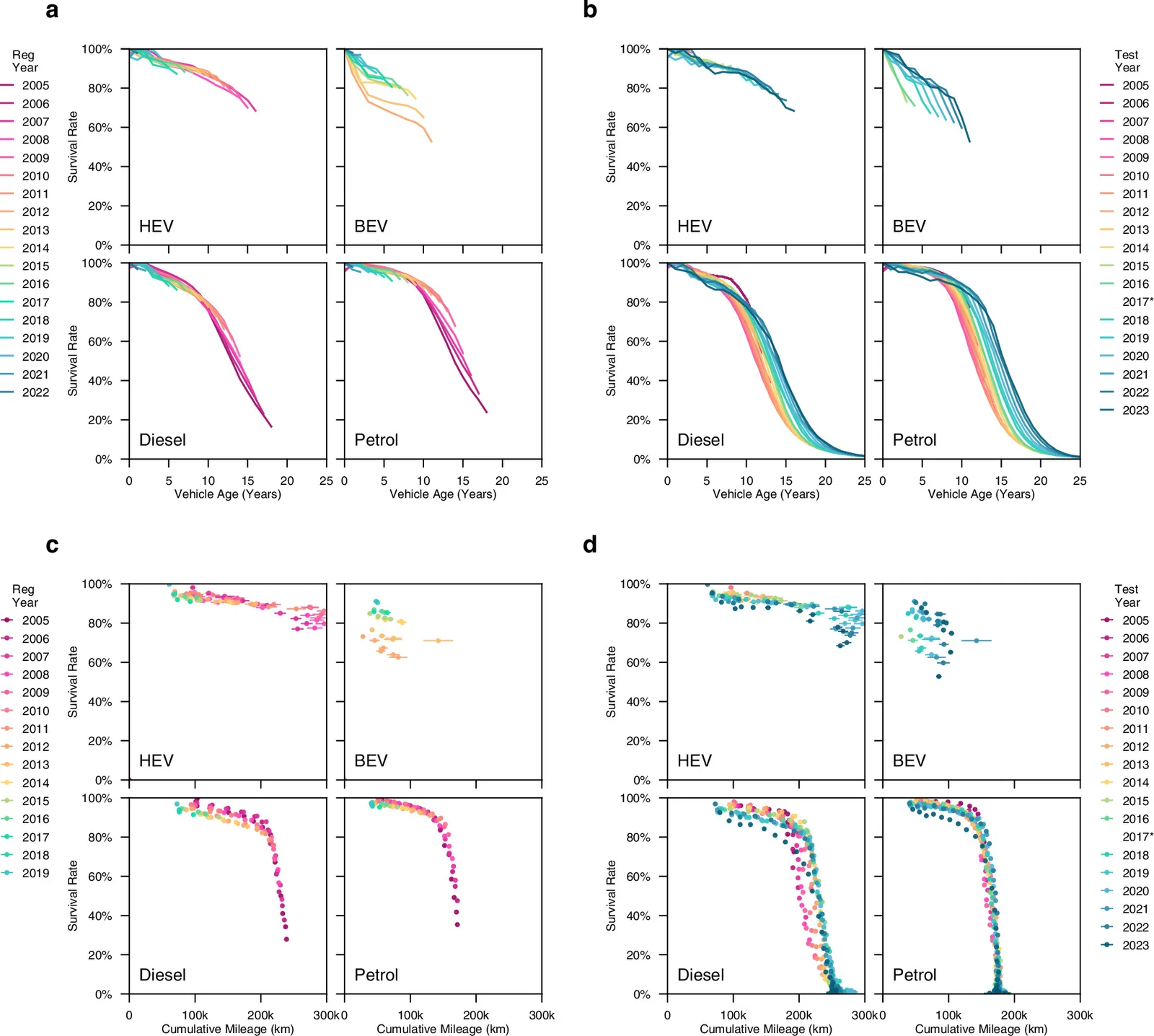
Vehicle survival rates in the UK as a function of age, mileage and type. The proportion of vehicles that remain in the fleet, known as the survival rate, by vehicle age (**a**, **b**) and by mileage (**c**, **d**). **a** gives survival rates for each registration year from 2005 to 2022 (thus showing the progression of each vehicle cohort over time); **b** provides the same relationship but taken across all registration years for the year the MOT test was taken (thus showing a snapshot of the fleet each year). **c**, **d** give the mean cumulative mileage of the vehicles exiting the fleet at each age from the curves given in (**a**, **b**) respectively, where lines show the standard error of the mean. Year 2017^*^ is not shown in panels (**b**, **d**) since the MOT data for 2017 has missing vehicle tests. Abbreviations: Battery Electric Vehicle (BEV), Hybrid Electric Vehicle (HEV), Registration Year (Reg Year). Source data are provided, giving sample sizes and underlying data for the figure.

We give these survival rates for each registration year from 2005 up to 2022 in Fig. 4a. Using MOT data allows us to also derive the mileage of vehicles that exit the fleet. The mileage recorded during the final roadworthiness test can also indicate the mileage of vehicles when they leave the fleet through scrappage or export; Fig. 4c shows these final test mileages at each age of the survival curve for each registration year. With this dataset, scrapped vehicles cannot be distinguished from exported vehicles that would continue to be used in another country. Therefore, our estimations of vehicle lifetime in years and mileage are a lower bound.

**Fig. 5:**
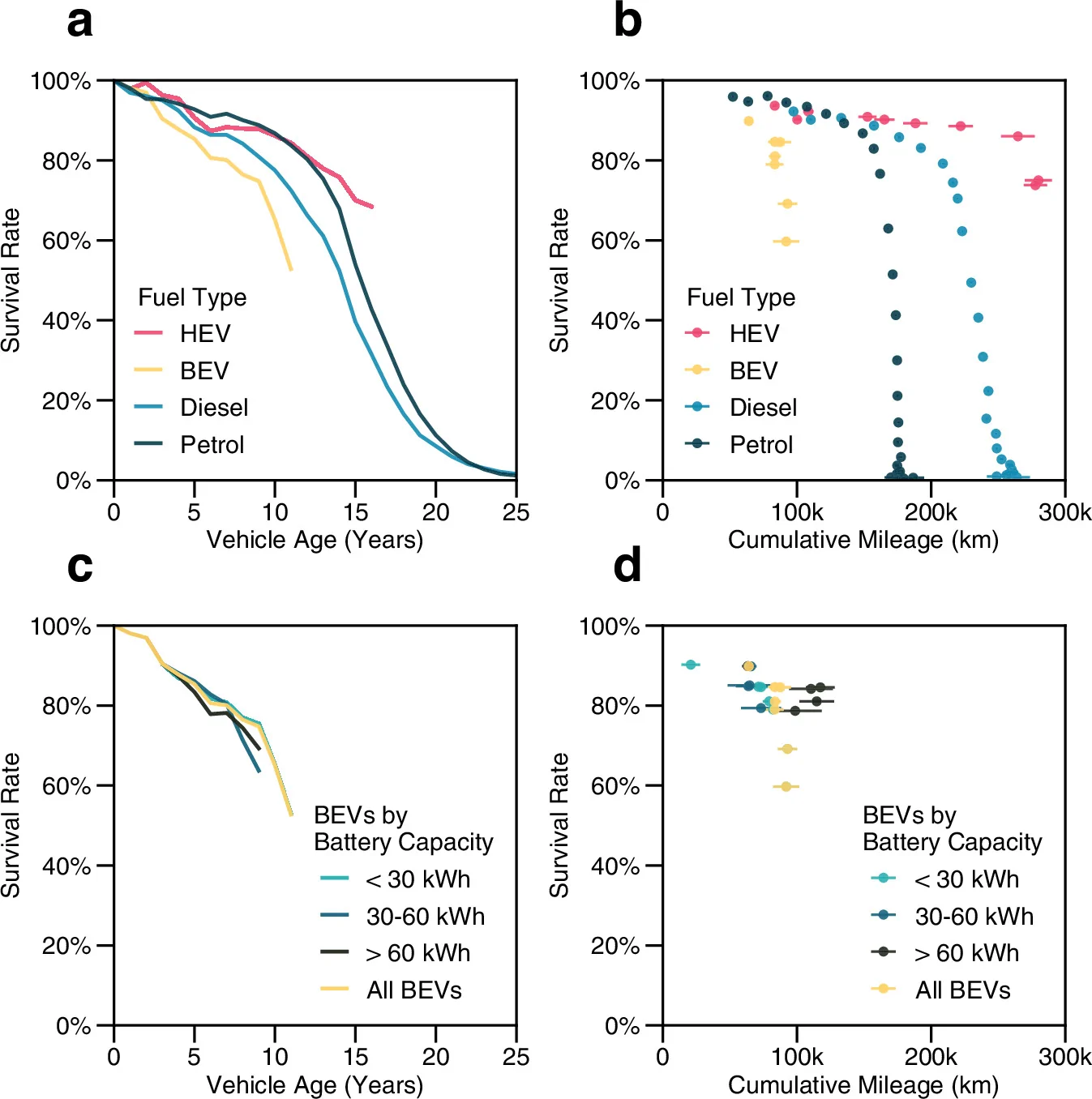
Vehicle survival rates in the UK by fuel type and battery capacity. The proportion of vehicles that remain in the fleet, known as the survival rate, by vehicle age (**a**, **b**) and by mileage (**c**, **d**); (**a**) gives survival rates across all registration years for 2023 (thus showing a snapshot of the fleet in 2023); (**b**) gives the mean cumulative mileage of the vehicles exiting the fleet at each age from the curves given in **a**, where lines show standard error of the mean. Panels (**c**, **d**) repeat (**a**, **b**), but disaggregate BEVs by battery capacity. Abbreviations: Battery Electric Vehicle (BEV), Hybrid Electric Vehicle (HEV). Source data are provided, giving sample sizes and underlying data for the figure.

Survival rates against age and mileage are given in Fig. 4b and Fig. 4d for the entire fleet across all registration years for each test year between 2010 and 2023, with Fig. 5 providing these relationships in 2023 by fuel type and BEV battery capacity. The trends in Fig. 4b indicate that all vehicle types are lasting longer, except for HEVs, where survival rates have not settled into a consistent trend. The median vehicle survival age, when 50% of vehicles have left the fleet, was 15.4 and 14.1 years for petrol and diesel vehicles in 2023, 3.8 and 2.8 years older than in 2010, respectively. The cumulative mileage at 50% survival rate was 170,000 km and 230,000 km for petrol and diesel cars, which aligns with values used for previous lifecycle analysis^6^, and has remained consistent across test years. The consistent lifetime mileages suggest that the recent trend towards lower annual mileage, as shown in Fig. 2, is directly contributing to the longer vehicle lifetimes in years. This shows that calendar age alone cannot accurately define vehicle lifetime, which is both dependent upon the individual vehicle technology and the wider fleet the vehicle is used in.

Historically, BEVs that have left the UK fleet did not last as long and were driven less than equivalent petrol or diesel cars exiting the fleet. We find that 47% of the first BEV cohort from 2012 had left the fleet by 2023 at age 11. This compares to 27, 16, and 16% of diesel, petrol, and HEVs from the 2012 cohorts, respectively. This discrepancy has reduced in newer model years, where for the 2019 cohort, 12% of BEVs have left the fleet, compared to 8, 6, and 5% for diesel, petrol, and HEVs, respectively. These shorter BEV lifespans are confirmed using the UK government’s online vehicle licensing statistics^25^, giving the number of licensed vehicles in the fleet each year, broken down by the registration year. This independent dataset provides 50% survival ages within ± 0.5 years of the MOT data; the survival curves in Fig. 4a, b are repeated using this alternative data in Supplementary Fig. 1. The current BEV and HEV survival rate curves do not yet follow Weibull distributions that are typically used to model vehicle survival^9,17,18,28^. This aligns with recent analysis suggesting UK vehicle survival rates are starting to deviate from typical Weibull curves for other vehicle types^9^. The mileage when BEVs and HEVs leave the fleet also do not follow the typical patterns of diesel and petrol cars, suggesting that accumulated mileage-related deterioration may not yet be the cause for these vehicles exiting the fleet.

The lifetime and final recorded mileage of BEVs are increasing each year, much like the annual mileage trends from in Fig. 2. The relationships in Fig. 4 show that 50% survival age and 50% survival mileage have both doubled from 2015 to 2023 test years. Since 2016, BEV survival age has linearly increased by 0.49 years per year (see Supplementary Fig. 2), reaching 11.2 years by 2023. If this linear trend continues, BEVs will match the diesel vehicle lifetime of 14.1 years by 2029. Since vehicles enter the national fleet data at three years of age, limiting the latest registration year to 2021, this implies BEVs sold from 2026 onwards are likely to have a comparable lifetime to petrol and diesel vehicles. The increase in BEV lifetime over time has not been consistent across model years. Subsequent model years have exhibited a step change in survival rates, as shown between BEV models from 2015 and 2016 in Fig. 2a. We find this phenomenon is repeated for the survival curves derived from both data sets (see Supplementary Fig. 1), but has not affected the recent consistent increase in survival from 2016.

When estimating vehicle survival rates in the first three years using the Vehicle Licensing Statistics dataset^25^, we occasionally observe small increases in the surviving cohort size at older ages. For example, Fig. 3a shows that 2014 and 2015 model year BEVs exhibit an apparent ~2% rise in survival between age two and age three. This counterintuitive increase occurs because the underlying number of licensed vehicles from these model years grew slightly. The licensing statistics only count vehicles that are currently taxed and on the road; they exclude vehicles that are untaxed, such as those held in dealerships on trade plates or temporarily legally declared off the road. When such vehicles are subsequently taxed and re-enter the licensed fleet, they reappear in the licensed total, creating the appearance of improved survival for that cohort, but these effects cannot be investigated separately using the publicly available data.

### Lifecycle greenhouse gas emissions

We modelled attributional lifecycle emissions by combining manufacturing emissions, based on vehicle mass and battery capacity, with use-phase emissions derived from MOT mileage data, and the GHG intensity of British electricity production. For BEVs, yearly average electricity emission intensities were applied, while for conventional and hybrid vehicles, emissions included fuel consumption and upstream supply chain impacts. We note that alternative ways to represent grid carbon intensity, such as short- and long-run marginal emissions, can yield different results than the average factors used here^29–32^.

**Fig. 6:**
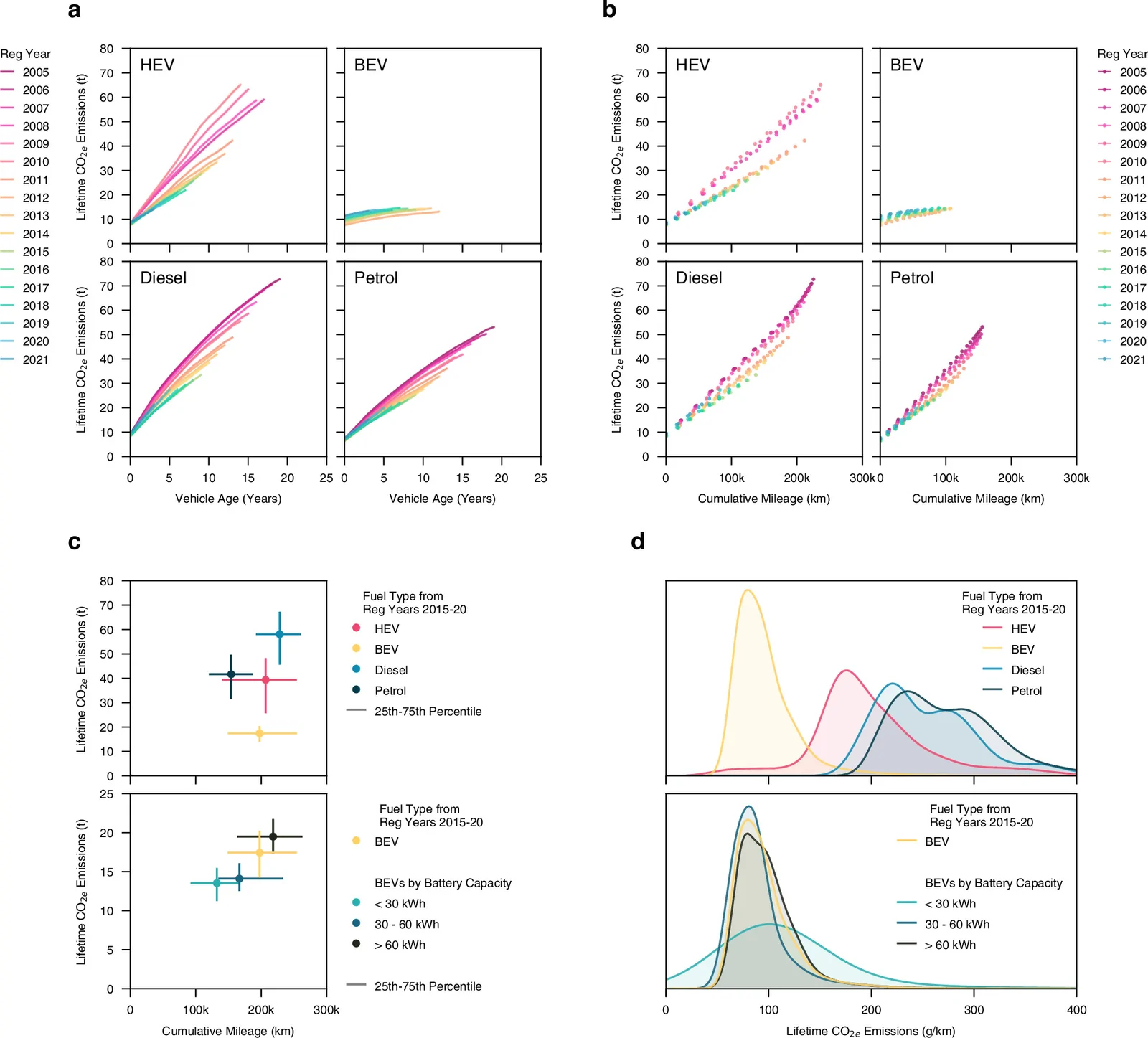
Historical and simulated lifecycle CO_2e_ emissions of existing vehicles. **a** gives the historical lifecycle emissions for vehicles in Britain’s fleet as a function of vehicle age, including the manufacturing emissions (the intercept on the vertical axis) and use-phase emissions (the slope of each line) up until the vehicle’s latest MOT test, where colour shows the registration year; (**b**) provides the same historical lifecycle emissions as a function of cumulative mileage at each vehicle age; (**c**) gives the median predicted whole-life lifecycle emissions using the projected mileage and survival using the transformer model for each fuel type and disaggregated by BEV battery capacity, where lines show the 25^th^ to 75^th^ percentiles; (**d**) the distribution of whole-lifetime emissions intensity for each fuel type and BEV battery capacity, calculated using the accumulated emissions and mileage from (**c**) emissions intensity can be thought of as the slope of a line from the origin to each point in panel (**c**). Abbreviations: Battery Electric Vehicle (BEV), Hybrid Electric Vehicle (HEV), Registration Year (Reg Year). Source data are provided, giving sample sizes and underlying data for the figure.

Figure 6a shows historical attributional lifecycle emissions for vehicles registered between 2005–2022, representing emissions from their point of manufacture through to their latest MOT test in 2025. Figure 6b shows the corresponding progression of emissions with the cumulative mileage at each vehicle age. Together, these show that newer cohorts of conventional and hybrid vehicles have lower attributed cumulative GHG emissions over time (comparing blue to magenta lines in Fig. 6a), which is in part due to improved fuel economy (comparing blue to magenta dots in Fig. 6b), and partly due to reduced utilisation. This trend is, however, reversed for BEVs, as newer cohorts with larger batteries have larger manufacturing emissions and are driven more intensively.

We find that older BEVs, which have now left the fleet, had lifecycle emissions of 228 g CO_2e_ per kilometre over their lifetimes, compared to 248 g CO_2e_ per km for HEVs, and 282–329 g CO_2e_ per km for petrol and diesel vehicles registered between 2012 and 2020. BEVs therefore had 9–31% lower attributed emissions per kilometre driven than HEV, petrol and diesel vehicles over their lifespan, despite the lower mileage and shorter lifetime of these older BEVs, and the higher carbon intensity of British electricity in the 2010s^29,33^.

To estimate attributional lifecycle emissions for current vehicles up until they exit the fleet, we used a transformer neural network to model the future mileage and survival of existing vehicles. This model simulated future MOT test results up until a vehicle exits the fleet, based upon the vehicle’s real-world MOT test history. The model was trained on MOT test histories of 1.14 million sample petrol and diesel vehicles, using mileage, age and fuel type as predictor variables. For annual mileage prediction achieved an R^2^ of 0.49 and MAE (mean absolute error) of 3600 km, and for vehicle survival prediction achieved an AU-ROC (area under the receiver operating characteristic curve) of 0.83 (please see the Supplementary Table 6 for full model validation metrics). The model was applied to a sample of 2.76 million active vehicles spanning 2005–2022 registration years, including all active BEVs in the fleet.

Figure 4c presents these projected lifetime attributional emissions against cumulative mileage for the different fuel types. We find that on average, current BEVs have projected attributional lifecycle emissions of 88 g CO_2e_ per kilometre, 63% lower than previous BEVs, which have already exited the fleet. In contrast, projected attributional lifetime emissions of current HEVs and conventional vehicles are projected to fall by only 18–24%, to 190 g CO_2e_ per kilometre for HEVs, and 254–270 g CO_2e_ per kilometre for petrol and diesel vehicles.

Compared to recent studies using UK-specific electricity generation emission intensities, we find our lifetime emissions for BEVs of 88 g CO_2e_ per kilometre falls between recent lifecycle analyses for the UK^3,34^. Our result is below Hill et al.^34^ (100 g CO_2e_ per km) and above Bieker^3^ (85 g CO_2e_ per km), with the differences primarily explained by battery manufacturing emissions and vehicle lifetime mileage. Hill et al. reported that battery size could affect their results by ± 5 g CO_2e_ per kilometre and that mileage could affect results by ± 17 g CO_2e_ per kilometre. With our mean battery capacity of 62 kWh, applying Hill et al.’s range of battery emission intensities (78–101 kg CO_2e_ per kWh) varies our results by ± 4 g CO_2e_ per kilometre. The remaining difference to Bieker arises mainly from embodied battery emissions: we assume a higher battery manufacturing intensity of 89 kg CO_2e_ per kWh (representative of vehicles manufactured for the UK market between 2012 and 2022) compared with their 65 kg CO_2e_ per kWh. In our study, vehicles had a larger mean battery size (62 kWh versus their 45 kWh), together with differences in assumed lifetime mileage. Battery manufacturing, therefore, contributes a substantial share of BEV lifecycle emissions, 5520 kg CO_2e_ or approximately 28 g CO_2e_ per kilometre over the vehicle’s lifetime, and is particularly sensitive to the assumed energy mix used to produce the batteries.

For BEVs, projected lifetime mileage varied depending upon battery size, reflecting the historical trend that lower mileage vehicles have had lower battery sizes. This resulted in lifetime emissions intensity being similar across the range of battery sizes. BEVs with battery capacities between 30 kWh and 60 kWh produce the lowest emissions at 84 g CO_2e_ per kilometre, and those with the highest battery capacities over 60 kWh produce 89 g CO_2e_ per kilometre.

## Discussion

A central uncertainty in vehicle lifecycle assessment is whether real-world electric vehicles are driven as intensively and for as many years as the conventional vehicles they are compared to. Using national records for the entire British fleet, including 0.5 million BEVs, we quantify how mileage and survival have evolved across vehicle types, and how this impacts attributional whole-life greenhouse gas emissions estimates. This evidence moves electrification debates from assumed to observed usage profiles, narrowing an empirical gap that has constrained LCAs.

Historically, BEVs were driven less intensively than conventional vehicles and did not survive as long in the British vehicle fleet. These trends have evolved rapidly as BEV technology has improved. Between 2012 and 2022, battery size increased from 21 kWh to 68 kWh, range increased from 120 km to 380 km, maximum charging speed increased from 44 kW to 140 kW, vehicle mass increased by 487 kg, and the most common body type shifted from hatchbacks (100%) to SUVs (52%). These combined technology shifts likely impact consumer attitudes^35^. Other factors beyond the vehicles themselves may also influence travel and purchase behaviour, such as the rapid increase in charging availability^14^.

We find that since 2017, new BEVs in Britain have been driven comparably to new conventional vehicles. Smaller-battery models equal the annual mileage of petrol, and larger-battery models equal that of diesel vehicles. By 2025, new BEVs and HEVs are approaching the usage of diesel cars, suggesting they now occupy a similar high-usage segment of the fleet. However, as BEV adoption grows, the usage trends we report may change as consumer behaviours evolve, and these behaviours cannot be forecast from this study alone.

As with mileage, BEV lifetimes have been increasing. For vehicles made from 2026 onwards, we expect the BEV lifetime to equal that of conventional vehicles if current trends continue. This supports consumers to make informed total cost of ownership decisions and environmental impact assessments^10^. This finding also validates a common simplifying assumption in life-cycle assessments^2–5^: it is now valid to model the current generation of BEVs with the same real-world usage as conventional vehicles.

Despite the low lifetime mileage of older BEVs, they still had lower attributed lifecycle greenhouse gas emissions than conventional vehicles. Previous LCAs have generally underestimated emissions from first-generation BEVs^2–5^ due to their smaller use-phase than conventional vehicles. Despite concerns about the additional emissions burden of manufacturing larger battery packs for long-range BEVs^36^, these models are currently driven more intensively than smaller BEVs, confirming preliminary evidence from the USA^11^. These data cannot determine whether consumers choose large-battery BEVs for their longer range, or whether high-mileage drivers adopt BEVs at higher rates. If these trends continue, the lifetime emissions intensity (gCO_2_e per kilometre) of large BEVs (such as the Tesla Model S and Jaguar I-Pace) is lower than that of small BEVs (such as the original Nissan Leaf and Renault Zoe). Mid-sized BEVs with 30–60 kWh batteries (such as the original Tesla Model 3) will have 6–23% lower emissions intensity than either small or large BEVs, as they provide the best trade-off between embodied emissions, lifetime mileage and energy consumption.

Our results suggest that BEVs registered in 2022–2026 may equal the lifetime mileage of petrol cars if current trends continue. Both usage and lifetime trends might partly reflect increasing battery capacity. Larger batteries enable longer journeys, making newer BEVs more versatile and thus used more. Battery degradation reduces usable capacity over time, further reducing range. As a simple thought experiment, acknowledging that real-world degradation varies substantially by chemistry and operating conditions, if capacity falls by 1.8–2.3% per year^37–39^, BEVs from 2012 would have lost up to 28% by 2024, exceeding the typical 20% end-of-life threshold^40,41^. Combining the 2012 cohort’s mean battery size (21 kWh) and energy consumption (5.9 km per kWh) gives an average range reduction from 124 km to 89 km, which might help explain why 47% of this cohort had exited the fleet by 2024. Smaller batteries are also cycled more intensively per kilometre driven, implying faster degradation^42^. The 2012 cohort had accumulated a mean of 725 equivalent full cycles by 2024, and by age 5 they reached 360 cycles, with only 71% remaining. In contrast, the 2019 cohort (with 59 kWh capacity and 330 km range) had accumulated only 208 cycles by the same age, and 93% remained in 2024. These differences reflect not only pack size but also technological advances between 2012 and 2019, which affect battery longevity. Future research could improve our vehicle specifications to provide the battery technology for each BEV, enabling a greater understanding for how battery technology effects overall longevity.

Annual mileage for HEVs was similar to diesel vehicles in earlier model years but is declining in more recent years. Both diesel and HEVs have lower fuel consumption than petrol vehicles, making them more economical for high-mileage users, including commercial applications such as Private Hire Vehicles (PHVs) and taxis. The high mileage observed for HEVs might therefore be explained by a larger proportion of these vehicles being used in PHV and taxi services. Based on the MOT data, we estimate that in 2015, 13% of the HEV fleet aged 3–8 years were PHVs or taxis, far exceeding the 2-3% share for petrol and diesel vehicles. In 2015, the annual mileage of HEV PHVs and taxis were approximately twice the mean for diesel and HEVs (39,500 cf. 16,500 and 19,200 respectively), and three times the average for petrol (11,200). By 2025, the share of HEVs used as PHVs or taxis had declined to 5%, although these vehicles continued to record similarly increased annual mileage as in 2015.

Policy incentives and consumer trends could have also influenced vehicle usage patterns. For example, exemptions from London’s congestion charge for HEVs and BEVs^43^ potentially encouraged higher mileage among these vehicles. In addition, the rising share of micro-HEVs and mild-HEVs, which provide smaller fuel-economy benefits than full hybrids^44,45^, could have contributed to the decline in mean annual mileage for the overall HEV category (which includes micro, mild and full hybrids). Future work could investigate these influences, but the latter would require more granular data on vehicle classification.

Similar to mileage, HEV lifetime mileage is also still changing. It appears to already exceed that of diesel vehicles at 230,000 km, but could be trending downwards. MOT data show that Toyota produced 92% of new HEVs in 2015, dropping to 28% by 2022 as more manufacturers entered the HEV market. The known longevity of Toyota vehicles^46^ may explain the higher lifetime mileage of earlier HEVs, which now is trending towards the fleet mean for petrol and diesel as the mix of manufacturers diversifies. The higher mileage of HEVs may also contribute to better longevity since vehicles will spend a larger proportion of their operation in hot-engine conditions.

Our results differ from those of Nguyen-Tien et al.^10^, who found longer BEV lifetimes than conventional vehicles using the same MOT dataset, for three reasons. First, by incorporating vehicle licensing statistics^25^ for the first three years of life, we show that historical BEV lifetimes are shorter than they reported^10^. Their derived survival rates do not align to our historically observed survival rates for all vehicles, with the largest discrepancy for BEVs, as shown in Supplementary Fig. 1. Second, our analysis accounts for declining annual mileage as vehicles age, rather than assuming constant mileage after the final recorded test. Ignoring this decline overestimates lifetime mileage. Third, we demonstrate that vehicle lifetime depends on both calendar age and cumulative mileage, with vehicles typically leaving the fleet after reaching similar lifetime mileages irrespective of how long this takes, which explains the observed increase in conventional vehicle lifespans over recent decades.

Our approach applies transformer neural networks to vehicle test data. This model was chosen because MOT test data comprises sequences of varying lengths, which suit transformer architectures for sequential inputs, and because the task requires simultaneous regression (predicting vehicle mileage) and classification (identifying whether a test represents the vehicle’s final record). The model could be improved in future by incorporating vehicle mass as a predictor of lifespan^10,18^ or integrating fuel consumption with fuel prices and weather conditions to forecast annual mileage^21^. Specific MOT test failure criteria could be added as predictor variables^16^, but were excluded here because they are often fuel-type specific and would reduce applicability to BEVs. Although more transparent approaches, such as multivariate linear regression, offer interpretable insights into relationships that are useful for policy applications^47^, our transformer model prioritises predictive accuracy for projecting vehicle usage and lifetimes. The model’s code is made publicly available to enable replication studies.

The MOT data covers vehicles while they are active in the fleet, so we assume vehicles exit through scrappage or export once they are no longer active. Public data do not identify which vehicles are sold internationally as second-hand exports. Using the ITF Used Vehicle Registration Database^48^, we find that ~ 15% of vehicles exiting the fleet are exported. The MOT data, therefore, underestimate true vehicle lifetime and mileage. Recent LCAs estimate that exported vehicles see 20–33% additional lifetime mileage beyond domestic licensing statistics, lowering lifetime CO_2e_ intensities by 7–22% for BEVs, 7–11% for HEVs and 4–8% for petrol and diesel vehicles^34,49^. Factoring this into our results would further increase the relative advantage of BEVs compared with conventional vehicles. We also exclude the emissions effects of second life applications for BEV batteries, which previous studies indicate could reduce BEV lifetime emissions by a further 1 g CO_2e_ per km^34^. It is therefore unplausible that vehicle exports and battery second life could change our overall conclusions: that BEVs provide lower lifecycle emissions per km than HEVs, petrol, and diesel cars.

The MOT data we used has reliability issues that should be addressed in future work. Mileage values include odometer fraud, where a vehicle’s lifetime mileage is illegally reduced to inflate its resale value, with an estimated 9.6% of used cars affected^50^. Some vehicles are also illegally unlicensed or missing a due MOT test, increasing from 0.6%^20^ to 1.2%^51^ over the decade to 2023. The prevalence of Statutory Off-Road Notices, which allow vehicles to be temporarily unlicensed and exempt from MOT tests if they are off the public road network, has doubled from 4% to 9% over the last decade^25^.

Our results show that vehicle calendar lifetime depends on the wider fleet they are part of. Over the last two decades, the number of cars in Britain has grown faster than vehicle kilometres driven, possibly marking the start of a “peak car” era^52^. Petrol and diesel cars are being used less intensively, especially at younger ages, while mileage at scrappage has not substantially changed. This suggests that fleet-wide trends are extending vehicle lifetimes. Previous life-cycle analyses avoid this issue by using mileage instead of calendar age to characterise lifetime, for example, assuming 170,000 km for petrol cars, which aligns with our observations^6^. Lifetime mileage is highly context specific: petrol cars last 333,000 km in the US compared to 120,000 km in China^6^. Repeating our analysis in other countries would help establish whether the usage and lifetime trends we identify are a global phenomenon.

Recent improvements in battery capacities and lower degradation rates might contribute to the longer lifetimes observed for newer BEVs. Broader trends in convenience and desirability might also influence usage patterns and scrappage decisions for reasons other than technical failure. The growing availability of public charging points in the UK, a 30-fold increase in the decade to 2025 reaching over 80,000 locations^14^, has increased the viability of longer journeys, and may explain the increased annual mileage observed. Across many sectors, consumers replace products more frequently when technology is advancing rapidly, then keep them longer as improvements become incremental. Smartphone lifetime increased from <2 to > 3.5 years^53,54^ between 2016 and 2023, a trend also seen with desktop computers^55^ since the slow-down of Moore’s Law^56^, and televisions since migrating to HD and 4 K resolution^55^. For passenger cars, most consumers are delaying the purchase of an EV until technology and price meet expectations^57–59^, reducing the value of first-generation BEVs that do not meet preferred performance criteria. Early BEVs might therefore have exited the fleet prematurely due to obsolescence compared to more advanced and affordable later models, rather than failure due to wear and tear.

This work shows that current-generation battery electric vehicles produce less than half the attributional lifecycle greenhouse gas emissions of conventional vehicles, based on real-world use by British drivers. This result required the rich administrative data available in the UK, which reveals how new technologies are used at a population scale. Strengthening MOT records (and international equivalents), for example, by adding BEV-specific attributes such as battery capacity and state-of-health^60^, would increase their scientific and policy value, allowing more questions to be answered as more BEVs enter the fleet. Better fleet instrumentation could allow real-world durability to be tracked and thus improved. Extending this approach to other countries would show whether the convergence of BEV mileage and lifetime is universal or dependent on specific technological, infrastructural, geographic or social factors. Finally, our lifecycle emission results are attributional, providing an account of current fleet emissions rather than the effect of specific actions. Future work could use causal or consequential methods to estimate how actions such as encouraging BEV adoption or altering scrappage patterns would affect emissions.

## Methods

### Data

The MOT data was taken from the UK government publicly available ‘Anonymised MOT tests and results’ for tests between 2005 and 2025^16^. The MOT test records contain the vehicle attributes (make, model, registration year, colour, etc.), vehicle lifetime mileage (odometer reading), test result, and a unique vehicle identification number that enables vehicles to be tracked over their lifetime. A random sample was drawn from the MOT dataset for conventional vehicles, comprising 5.9 million diesel vehicles and 6.1 million petrol vehicles from their respective total populations of 21.1 million and 40.1 million, respectively. The complete sets of hybrid electric vehicles (HEVs) and battery electric vehicles (BEVs) in the MOT database were included in the analysis, contributing 1.1 million and 0.5 million vehicles, respectively. In total, 12 million vehicles across all fuel types were analysed. This substantial sample size ensured statistical robustness, with sufficiently small standard errors for the mean values reported in our results, such that further expansion of the sample would not materially alter our findings. The processing steps to achieve these samples are outlined in the Supplementary Note 1.

We used three vehicle database sources to provide other vehicle attributes not contained in the MOT data. The first database from the European Environment Agency’s CO_2_ monitoring data^23^ was used to estimate fuel consumption and vehicle mass using the methods from Newman et al. (2024)^19^ for diesel, petrol and HEV vehicles. This data includes vehicles registered between 2010 and 2022. For vehicles registered prior to 2010, the second database was used from the UK’s Vehicle Certification Agency^22^ to estimate fuel consumption for vehicles made between 2001 and 2010 (vehicle mass was not included in the database) using the same methods. Vehicles registered before 2001 were assumed to have a 2001 model year for fuel consumption estimates, and vehicles registered before 2010 were assumed to have a registration year of 2010 for mass estimates. This method used the vehicle’s cylinder capacity, fuel type, and model year to create a model to predict the fuel consumption per kilometre. We differed from Newman et al.^19^ by using Random Forest Regressors with the Python package scikit-learn^61^, achieving comparable model performance of *R*^2^ = 0.85 for fuel consumption estimates and *R*^2^ = 0.89 for mass estimates. The fuel consumption values given in the two databases are type-approval values, measured on official driving cycles which have been shown to be biased, and so we estimated real-world values using correction factors from Hill et al.^34^ that are given in Supplementary Table 6.

The third database gave vehicle attributes for BEVs, providing battery capacity in kWh and energy consumption in kWh per km^13^. The database provides energy consumption for real-world conditions for BEVs operating in European driving and weather conditions, where upstream losses from charging were considered separately (see Lifecycle greenhouse gas emissions section below). We include BEVs models from 2012 to 2022, thus spanning periods where NEDC and WLTP drive-cycles were used to calculate energy consumption. The EV database uses these reported drive-cycle values to provide a consistent estimation of real-world BEV energy consumption across this period^62^. Vehicles in the MOT data were matched to the vehicles given in the EV database using the make, model, and registration year. The MOT data did not include enough information to match each vehicle uniquely to the EV database (e.g., where multiple variants of the same make and model exist), and so some vehicles in the MOT were assigned the mean attributes of the corresponding matched vehicles in the EV database.

To calculate the survival rates using the cohort approach, we assumed that each vehicle’s final recorded test was the point when the vehicle exited the fleet. However, since all active vehicles will have a final test in 2024 or 2025, the last two years of the MOT data, we employed the criteria from Nguyen-Tien et al. (2025)^10^ to determine if a vehicle has left the fleet for the final two years of the MOT data. Our methods required a “buffer period”, a period after the last MOT test, of 24 months, compared to their range between 15 and 21 months. Because of this buffer period, our survival curves were calculated up until 2023: vehicles with a test in 2023 but no test in 2024 or 2025 were assumed to have exited the fleet.

The first cohort for BEVs was the 2012 registration year since the population of older vehicles was too small to follow consistent mileage patterns (3000 BEVs were registered before 2012). Likewise, the first cohort for HEVs in 2007 was chosen for the same reason (17,000 HEVs were registered before 2007). The oldest registration year considered for diesel and petrol cohorts was 1970.

Most vehicles operating as a private hire vehicle or taxi require an MOT test frequency of 6 months^63^. To produce an approximation of the population of private hire vehicles and taxis, we filter the MOT data for vehicles that have had a test within 0.4 to 0.6 years. This filtering step will include a portion of vehicles that coincidentally have this time between MOT tests. Some local authorities have differing rules regarding MOT test frequency, and so we cannot identify all taxis and private hire vehicles with this method.

### Lifecycle greenhouse gas emissions

We calculated vehicle attributional lifecycle emissions by combining historical and projected vehicle mileage from MOT data with vehicle attributes from databases and emission parameters from the literature.

Manufacturing emissions were calculated using emission factors based on vehicle mass and battery capacity. For battery electric vehicles (BEVs), we separated battery mass from non-battery mass using a battery pack energy density of 7 kg per kWh^64^. Non-battery components of BEVs were assigned manufacturing emissions of 4.7 kg CO_2e_ per kg vehicle mass, whereas petrol, diesel and HEVs were assigned 5.2 kg CO_2e_ per kg vehicle mass^3^. BEV battery manufacturing emissions were calculated at 89 kg CO_2e_ per kWh with a plausible range from 78 to 101 kg CO_2e_ per kWh^34^. For HEVs which lacked battery size data in vehicle attributes, we applied the same manufacturing emissions rate as conventional vehicles, 4.7 kg CO_2e_ per kg (we do not include Plug-in HEVs in this study). This assumption would underestimate 0.13 tCO_2e_ (2%) of manufacturing emissions for a HEV with a 2 kWh battery and 2000 kg mass.

For BEV use-phase emissions, we multiplied energy consumption by electricity emission intensity. We derived yearly generation mixes from 2012 to 2025 using methods from Staffell (2017)^65^ and projected to 2050 using the mean of two electrification net-zero pathways from the UK’s NESO (2024)^66^. Combining these mixes with attributional lifecycle emission factors for each generator type^67^ yielded yearly CO_2e_ per kWh intensities, which were assigned based on MOT test years. Vehicle energy consumption was adjusted to account for transmission and distribution losses, 7.5%^65^, and charging losses, 10%^68^. For petrol, diesel, and HEV vehicles, use-phase CO_2e_ emissions were calculated from fuel consumption, with upstream supply chain emissions contributing 10.5%^69^. The average emissions intensity of hydrocarbon fuels was modelled as remaining static in future years.

Following the recommendations in ISO 14040/14044 to use the appropriate emission factor accounting method for the lifecycle analysis research aim, we used average emission intensity rather than a short- or long-run marginal emission intensity^30–32^. This decision was due to our study providing an attributional lifecycle analysis for vehicles already in the British vehicle fleet and not assessing consequences from decisions. By using yearly averages for electricity emissions intensity, we note that historical CO_2e_ emissions up to 2020 may be underestimated by up to 5% due to the current coincidence of real-world charging profiles and the diurnal pattern of electricity emission intensity^29^.

### Predicting future mileage and survival

Transformer neural network models^70,71^ perform well for tasks using sequences of data of varying length, making them a natural fit for our application of predicting future tests for individual vehicles based on their real-world MOT history. We created a simple Transformer model using the PyTorch library^72^ to use the MOT test history of an individual vehicle to predict the mileage and probability of vehicle survival for the vehicle’s next test (that the next test will be the vehicle’s last). The model’s complexity was minimised for this application, using a limited number of neurons and an encoder-only architecture, to allow for smaller computing requirements and greater accessibility. The model specification is outlined in the Supplementary Note 3.

The model was trained on 9 million real-world test sequences from a sample of 1,139,000 vehicles, consisting of 709,000 petrol and 430,000 diesel vehicles (multiple sequences were taken from each vehicle’s MOT test history depending on their length). The training sample was selected based upon the survival curves presented in Fig. 3b. The proportion of vehicles leaving the fleet from the survival curve at each age was used to determine the proportion of vehicles in the training sample with final tests at that age, thus producing a sample of vehicles with survival rates matching the real-world distribution. The training objective encompassed both regression (mileage prediction) and classification (survival probability prediction) tasks. The model does not predict any other test metrics, such as vehicle colour or manufacturer, and so these metrics are assumed to be constant from the last test record onwards.

The model was validated using a split test, where 80% of the sequences were randomly selected to train the model, and the remaining unseen out-of-sample data was used for model validation. The performance of the mileage regression prediction is illustrated in Supplementary Fig. 3, and the performance metrics of the mileage regression and survival probability classification are given in Supplementary Table 6. This validation reveals the model performs robustly on unseen data. The mileage predictions exhibit some systematic bias, overestimation for low-mileage vehicles and underestimation for high-mileage vehicles (Supplementary Fig. 3c). This pattern of regression toward the mean is a well-documented and inherent property of machine learning regression models arising from the bias-variance trade-off and has been shown to be common across diverse ML architectures rather than specific to any particular model^73^. As this bias is approximately symmetric about the mean, the overestimation and underestimation largely cancel at the fleet-aggregate level, and the aggregate errors are modest relative to observed variability and do not materially affect fleet-level mileage and lifetime and the upstream emissions estimates.

Based upon the observed trends in Figs. 2–5, which demonstrate that BEV and HEV mileage and lifetimes are converging toward those of petrol and diesel vehicles, we train the model on petrol and diesel vehicles and apply the learned patterns to BEVs and HEVs. For example, high-mileage BEVs, often equipped with larger batteries, exhibit usage trends similar to diesel vehicles, while lower-mileage BEVs with smaller batteries align more closely with petrol vehicle usage patterns. This provides an estimation of BEV and HEV mileage and lifetime beyond the currently available MOT data and should not be interpreted as predicting real-world values. As the MOT data does not yet include the full lifetimes of BEVs and HEVs, the training data included only petrol and diesel vehicles. For projections, each BEV and HEV was classified as either petrol or diesel based on its cumulative mileage at the last observed MOT test, using a cutoff value midway between the typical mileages of petrol and diesel vehicles (detailed in the Supplementary Note 3).

To validate the model’s performance on BEVs and HEVs, we conducted a leave-last-value-out test. For each HEV and BEV sequence for vehicles aged 4 and over, the final real-world MOT test record was withheld, and the model was used to predict both annual mileage (regression) and survival probability (classification) for that held-out test using only the preceding records. Prediction performance on these withheld BEV and HEV tests was comparable to the results obtained on petrol and diesel vehicles, and are given in Supplementary Table 7. This evaluation on the most recent tests was appropriate because mileage and survival trends for BEVs and HEVs are converging toward those of petrol and diesel vehicles in recent years (Figs. 2–5). These results confirm that the model is suitable for projecting BEV and HEV mileage, survival, and emissions.

### Reporting summary

Further information on research design is available in the Nature Portfolio Reporting Summary linked to this article.

## Supplementary information

Supplementary Information

Reporting Summary

Transparent Peer Review file

## Data Availability

The data^74^ used in this study are available at https://doi.org/10.5281/zenodo.21870456. Source Data for each figure are provided in this data. Each of the underlying datasets used in the study are publicly available. The dataset of MOT test results is available at https://www.data.gov.uk/dataset/e3939ef8-30c7-4ca8-9c7c-ad9475cc9b2f/anonymised_mot_test. The UK’s Vehicle Certification Agency dataset providing fuel efficiency is available at https://carfueldata.vehicle-certification-agency.gov.uk/. The European Environment Agency’s CO_2_ monitoring dataset, providing fuel efficiency and vehicle mass, is available at https://www.eea.europa.eu/en/datahub/datahubitem-view/fa8b1229-3db6-495d-b18e-9c9b3267c02b. The BEV vehicle attribute data can be found at https://ev-database.org/.

## Code availability

Code^75^ is available from: https://doi.org/10.5281/zenodo.22098382. We intend to update the public repository with revised mileage and survival estimates as new MOT data releases become available at: https://github.com/DanielMehlig/ev-mileage-and-lifetime.
