# Supplementary material for: Real-world mileage and survival of electric vehicles in Britain and implications for lifecycle emissions
Source: Nat Commun. 2026 Sep 21;17:10989. doi: 10.1038/s41467-026-77892-4 (PMC13683361; doi:10.1038/s41467-026-77892-4)
Supplement: Supplementary file 1 [file 41467_2026_77892_MOESM1_ESM.pdf]

Supplementary Information:

Real-world mileage and survival of electric vehicles in Britain and implications for lifecycle emissions

Daniel Mehlig[1*] and Iain Staffell[1]

[1] Centre for Environmental Policy, Imperial College London, London, UK.

* Corresponding author d.mehlig18@imperial.ac.uk

Supplementary Items List

Supplementary Notes:	Page Number
**Supplementary Note 1.** MOT Data Filtering Steps and Sample Sizes.	3
**Supplementary Note 2.** Transformer Model Training Data Sample.	9
**Supplementary Note 3.** Transformer Model Specification	11
**Supplementary Figures:**	
**Supplementary Figure 1**. Validation of vehicle survival curves through an alternative data source.	7
**Supplementary Figure 2.** BEV 50% vehicle survival age between 2016 and 2023.	8
**Supplementary Figure 3.** Model validation of predicted annual vehicle mileage.	14
**Supplementary Tables:**	
**Supplementary Table 1.** Light Goods Vehicle make and models excluded during initial filtering. steps	5
**Supplementary Table 2.** Sample sizes after sequential filtering steps.	6
**Supplementary Table 3.** Transformer training data sample sizes after sequential filtering steps.	10
**Supplementary Table 4.** Transformer training data sample sizes after sequential filtering steps.	10
**Supplementary Table 5.** Mileage cutoff for BEVs and HEVs.	13
**Supplementary Table 6.** Model validation of mileage and survival prediction.	15
**Supplementary Table 7.** Model validation of mileage and survival prediction for BEV and HEV leave-last-test-out evaluation.	16
**Supplementary Table 8.** Adjustment factors for type-approval fuel consumption to real world.	17
**Supplementary References**	18

Supplementary Note 1. MOT Data Filtering Steps and Sample Sizes

The MOT dataset required preprocessing before it could be used for analysis. Supplementary Tables 1-2 summarise the sequential filtering steps applied and the resulting sample sizes.

Only vehicles with passenger car test classes (class 4) were used from the MOT data. Some Light Goods Vehicles are within this test class, and so previous studies using the MOT data excluded these vehicles using a set of vehicle make and model combinations[1,2]. We use this approach by filtering out the make and model combinations given in Supplementary Table 1 below. These vehicles were removed before the filtering steps below were applied to passenger cars. Other vehicle types, such as Motor Tricycles, are also included within test class 4 and were not excluded in this analysis due a lack of make and model combinations. Future usage of this MOT dataset could improve upon this limitation, although their number is small and will not significantly alter the findings of this study.

Mileage and Survival Sample

The following sampling and filtering steps were taken to produce the sample for the mileage and scrappage analysis. The corresponding sample sizes for each step are given in Supplementary Table 2.

Starting Sample: We began with the complete MOT dataset containing all passenger cars from 2005 to 2025 MOT tests.

Random Sample Selection: We selected a random sample of vehicle IDs, including 1 million vehicles from test years 2005-2006 across all vehicle ages, and 200,000 vehicles from each subsequent test year for vehicles aged 3 years (the age most vehicles enter the MOT data). For BEVs and HEVs, due to their smaller population sizes, all available vehicles were retained.

Vehicle Attribute Assignment: Vehicles were assigned attributes based upon the MOT data. For conventional vehicles, this included fuel consumption data from the Vehicle Certification Agency and vehicle mass data from EEA CO_2 monitoring records, where vehicles without valid ‘cylinder_capcity’ records we removed. For BEVs, vehicles were matched to the EV Database to obtain battery capacity and energy consumption values. Vehicles that could not be successfully matched or were erroneously classified (e.g., PHEVs incorrectly labelled as BEVs) were excluded.

Data Error Removal: Records with fundamental data errors, such as missing values, non-numeric entries, or logically impossible values were removed.

Valid Mileage Between Tests: We calculated the mileage increment between consecutive tests and removed vehicles with implausible values, which could indicate odometer errors or data entry mistakes.

Valid Mileage Per Year: Mileage per year was calculated by dividing the mileage between tests by the time between tests. Vehicles with invalid values for this metric were excluded.

Valid Time Between Tests: Records with implausible time intervals between tests were removed from the dataset.

Minimum Annual Mileage: Based on previous studies using the MOT dataset[1,2], vehicles with recorded annual mileage below 100 miles (161 km) were excluded as these likely include data entry errors.

Supplementary Table 1. Light Goods Vehicle make and models excluded during initial filtering steps of the Ministry of Transport (MOT) data.

**Make**	**Model**
Ford	Transit
Mercedes	Sprinter
Mercedes	Vito
Vauxhall	Vivaro
Vauxhall	Combo
Vauxhall	Movano
Volkswagen	Transporter
Volkswagen	Caddy
Citroen	Relay
Citroen	Dispatch
Citroen	Berlingo
Peugeot	Partner
Peugeot	Boxer
Renault	Trafic
Renault	Kangoo
Fiat	Ducato

Supplementary Table 2. Sample sizes after sequential filtering steps where samples were used to assess milage and vehicle survival. Abbreviations: Battery Electric Vehicle (BEV), Hybrid Electric Vehicle (HEV), Identification (ID).

**Filtering Steps**	**Diesel**	**Petrol**	**HEV**	**BEV**
1. Starting Sample	21,121,692	40,097,585	1,128,650	502,064
2. Random Sample of Vehicle IDs	5,883,823	6,118,226	1,128,650	502,064
3. Vehicle Attributes	5,879,176	6,116,137	1,127,429	502,064
4. Missing Test Mileage	5,391,417	5,686,933	1,092,648	494,524
5. Mileage Between Tests	5,130,304	5,453,975	1,082,832	493,363
6. Mileage per Year	5,125,801	5,451,107	1,076,780	493,358
7. Time Between Tests	5,125,771	5,451,094	1,076,746	493,358
8. Less than 100 Miles per Year	5,125,653	5,450,926	1,076,742	490,376
**Final Sample**	**5,125,653**	**5,450,926**	**1,076,742**	**490,376**

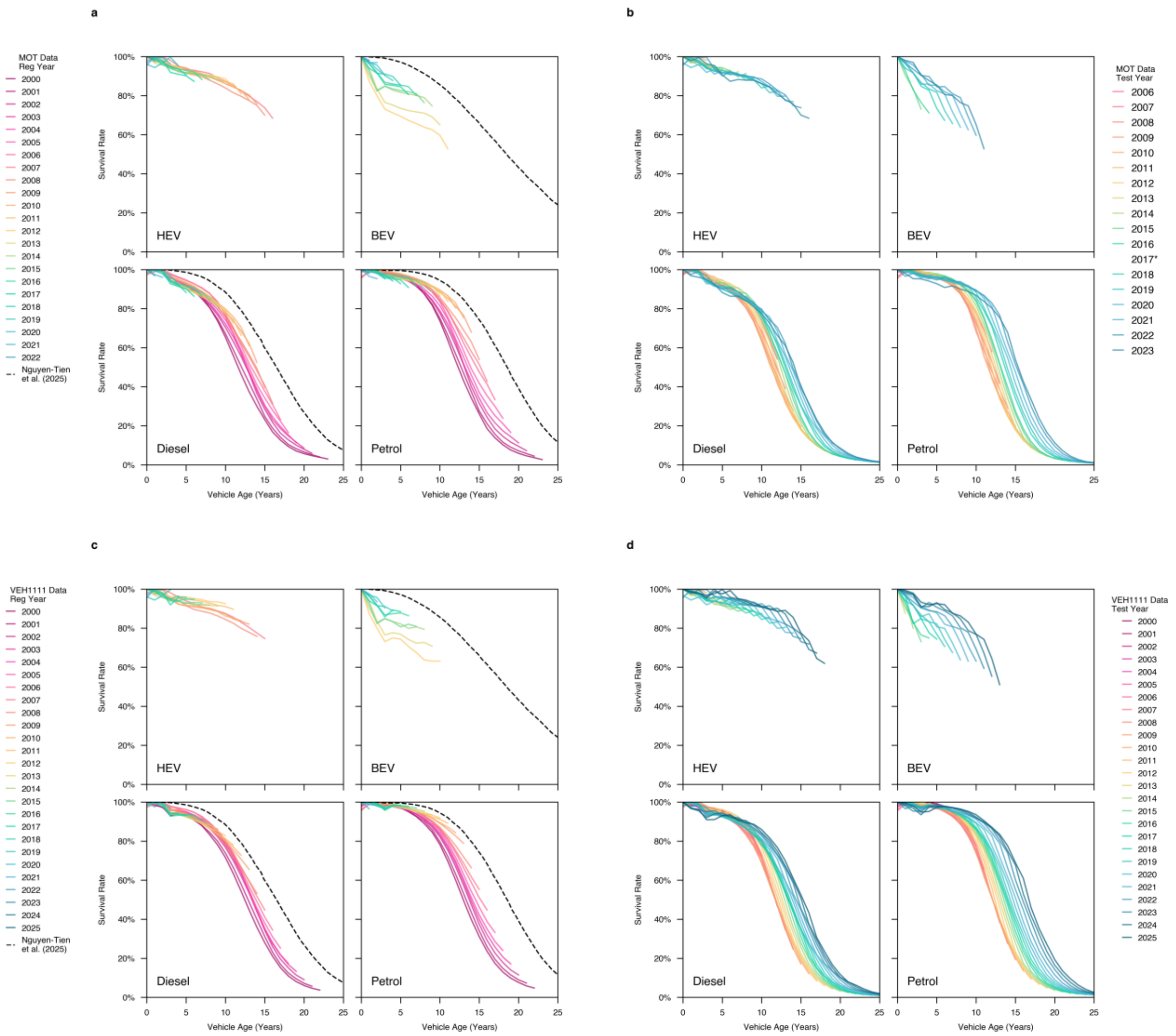

Supplementary Figure 1. Validation of vehicle survival curves through an alternative data source. Panel a gives the survival rates by age for each of vehicle registration year, repeating the panel given in Figure 4a; panel b gives survival rates by age for each year of the MOT data, repeating the panel given in Figure 4b; both these panels are repeated using licensed vehicle numbers from the VEH1111 data table from UK Department of Transport's online vehicle licensing statistics[3] that can provide additional years of vehicle survival, up to 2025. Survival curves given in dashed-black in panels a and c were taken from Figure 2 of Nguyen-Tien et al.[4]. Abbreviations: Battery Electric Vehicle (BEV), Hybrid Electric Vehicle (HEV), Ministry of Transport (MOT), Registration (Reg).

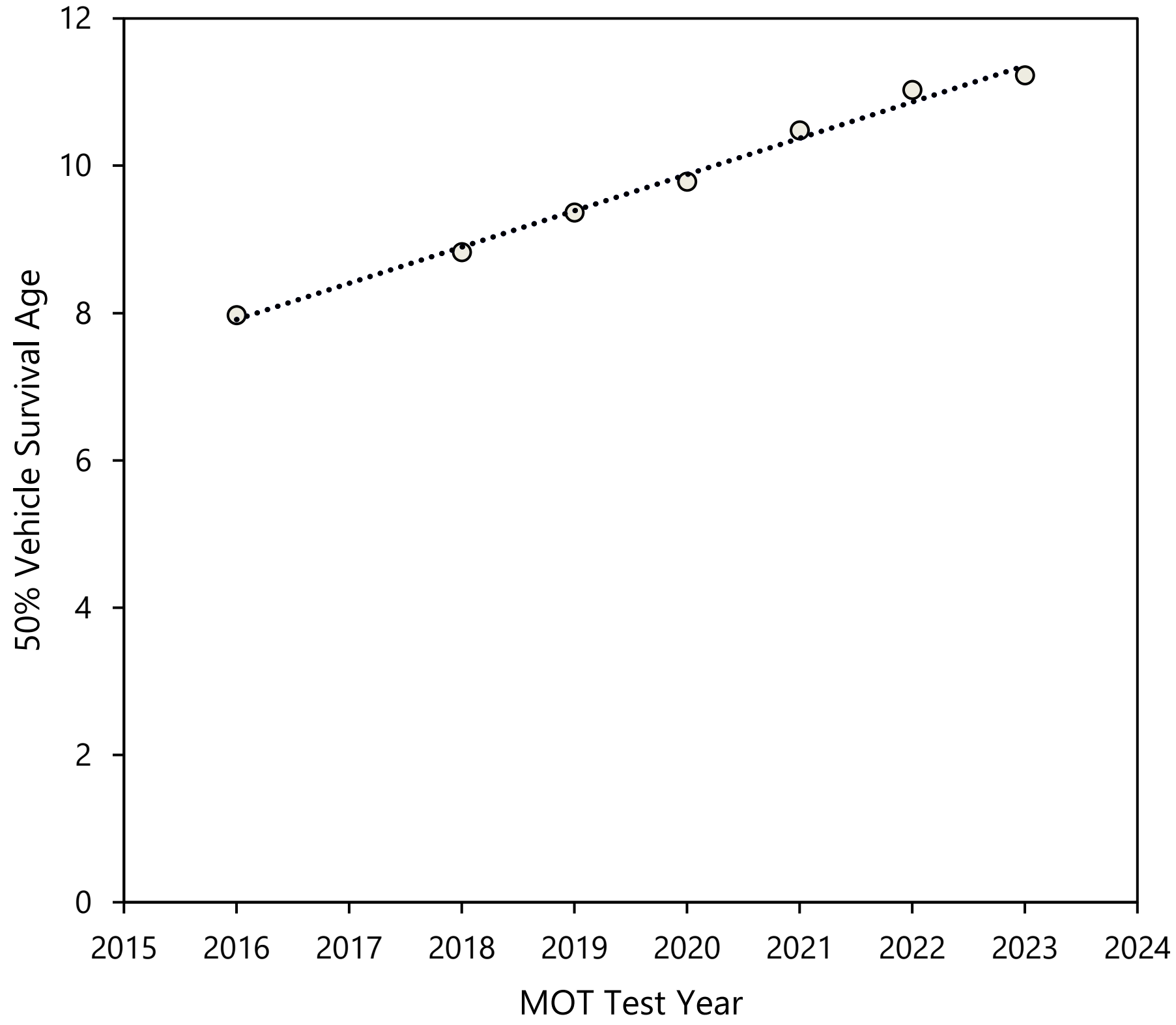

Supplementary Figure 2. BEV 50% vehicle survival age between 2016 and 2023. Data points taken from the 50% vehicle survival age from Figure 2 b, with linear regression line of best fit showing an increase of 0.49 years per year (R^2=0.99). Abbreviations: Battery Electric Vehicle (BEV), Ministry of Transport (MOT).

Supplementary Note 2. Transformer Model Training Data Sample

The following sampling and filtering steps were taken to produce the training data sample for the transformer neural network model. The corresponding sample sizes for each step are given in Supplementary Table 3.

Time between tests greater than 2 years: Vehicles with excessive time between tests were removed from the training data to improve the model's predictions.

Mileage per Year greater than 100,000 miles (161,000 km): Vehicles with excessive mileage between tests were removed from the training data to reduce the impact of extreme outliers.

The sample was constructed to match the survival rate curves observed in the 2022 data (Figure 3C in the main text) for petrol and diesel vehicles. This sampling approach ensured that the model was trained on a representative distribution of vehicle lifespans. HEVs and BEVs were excluded from the training data as they do not yet have complete lifespan data available in the MOT records.

Transformer Model Prediction Data Sample

The following sampling and filtering steps were taken to produce the prediction data that the transformer neural network model used to predict future vehicle usage and survival. The corresponding sample sizes for each step are given in Supplementary Table 4.

Time between tests greater than 2 years: Vehicles with excessive time between tests were removed from the training data to improve the model's predictions.

Vehicles with less than 3 Test Records: The Transformer model was designed to take a minimum of 3 sequences to make a prediction, therefore remove vehicles with less than 3 test records.

Mileage per Year greater than 100,000 miles (161,000 km): Vehicles with excessive mileage between tests were removed from the training data to reduce the impact of extreme outliers.

Random Sample Selection: Given the computational constraints of simulating the future of the entire sample, we selected a stratified random sample of vehicle IDs, including 50 thousand vehicles from each registration year between 2005-2022. For BEVs, due to the smaller population size, all available vehicles were retained from these registration years.

The sample was constructed to match the survival rate curves observed in the 2023 data (Figure 3C in the main text) for petrol and diesel vehicles. This sampling approach ensured that the model was

trained on a representative distribution of vehicle lifespans. HEVs and BEVs were excluded from the training data as they do not yet have complete lifespan data available in the MOT records.

Supplementary Table 3. Transformer training data sample sizes after sequential filtering steps.

Transformer Training Data	Diesel	Petrol
Starting Sample	4,986,712	5,238,346
1. Time between tests >2 Years	4,647,347	4,870,532
2. Mileage per Year > 100,000	4,640,061	4,867,176
3. Fit Sample to Survival Rate	429,753	708,824
**Final Sample**	**429,753**	**708,824**

Supplementary Table 4. Transformer prediction data sample sizes after sequential filtering steps. Abbreviations: Battery Electric Vehicle (BEV), Hybrid Electric Vehicle (HEV), Identification (ID).

Transformer Training Data	Diesel	Petrol	HEV	BEV
Starting Sample	4,986,712	5,238,346	1,069,226	490,243
1. Time between tests >2 Years	4,647,347	4,870,532	1,041,320	484,161
2. Vehicles with less than 3 records	4,372,405	4,563,632	1,041,120	484,161
3. Mileage per Year > 100,000	4,366,198	4,560,971	1,040,700	484,120
4. Random Sample of Vehicle IDs (2005 to 2020)	449,960	449,948	292,260	484,120
**Final Sample**	**449,960**	**449,948**	**292,260**	**484,120**

Supplementary Note 3. Transformer Model Specification

Model Architecture and Training

The model processes sequences of MOT test data using the following features for each test: fuel type (categorical), final test (categorical), annual mileage, total mileage, vehicle age, and time between tests.

The transformer model architecture was implemented using PyTorch, consisting of an encoder-only design to predict both future mileage (regression) and vehicle survival probability (classification). The encoder-only architecture was chosen for its computational efficiency and the nature of the prediction task. The aim is to encode the sequence of MOT tests into a fixed-length representation from which predictions about the next test can be made, rather than generating a sequence of future predictions. While a decoder could potentially enable multi-step predictions in future work, the current approach prioritises robust single-step predictions with lower computational requirements.

Model Components

The transformer encoder consists of an input embedding layer with a model dimension of 128 (input_dim → d_model = 128), a positional encoding layer with a maximum sequence length of 25, six transformer encoder layers, eight attention heads per layer, a hidden dimension of 256 in the feed-forward networks, and a dropout rate of 0.1 for regularisation.

The model architecture and hyperparameters were selected through a combination of computational constraints and validation performance. The encoder-only design with relatively small dimensions (d_model = 128, dim_feedforward = 256) allowed the model to run efficiently on modest consumer hardware while maintaining strong predictive performance. This design choice facilitates broader accessibility and potential deployment in resource-constrained environments. The model uses two separate prediction heads that combine linear layers with a rectified linear unit (ReLU) activation function and randomly drop 10 % of activations: the mileage prediction head follows the sequence Linear → ReLU → Dropout(0.1) → Linear, while the survival prediction head follows Linear → ReLU → Dropout(0.1) → Linear → Sigmoid.

Training

Training used a batch size of 15 000 sequences and a learning rate of 0.001 with the Adam optimiser. Learning-rate scheduling was performed with ReduceLROnPlateau (patience = 2). The loss function combined mean squared error (MSE) for mileage and binary cross-entropy (BCE) for survival, weighted in a 1 : 2 ratio (mileage : survival). Gradient clipping was applied with a maximum norm of 1.0, and early stopping was based on validation loss. The hardware comprised an Intel i7 CPU (4 cores, 8 threads), 32 GB of RAM, and an Nvidia GPU with 8 GB GDDR5 RAM and 1920 CUDA cores.

Data Processing

Sequences were processed with a minimum length of 3 tests and a maximum length of 8 tests. Features were standardised using StandardScaler and categorical variables were encoded with LabelEncoder. An 80/20 randomised train/test split was used, with validation performed via chronological holdout.

BEV and HEV Classification

For prediction purposes, BEVs and HEVs were classified as either petrol or diesel based on their lifetime mileage at their final real-world test. The classification threshold was determined for each vehicle registration year as the midpoint between the mean final test mileage of petrol and diesel vehicles. This approach ensures that high-mileage BEVs and HEVs are treated similarly to diesel vehicles in predictions, while lower-mileage ones are treated similarly to petrol vehicles, reflecting the observed convergence in usage patterns. These mileage thresholds for each registration year are given below in Supplementary Table 5.

Supplementary Table 5. Mileage cutoff for BEVs and HEVs, where vehicles with latest test mileage greater than or equal to this threshold are categorised as diesel and below as petrol.

Registration Year	Test Mileage Threshold (miles)
**2005**	116557
**2006**	115871
**2007**	115131
**2008**	111952
**2009**	106846
**2010**	103727
**2011**	99737
**2012**	93080
**2013**	86388
**2014**	80307
**2015**	74246
**2016**	67408
**2017**	61304
**2018**	55292
**2019**	47847
**2020**	40053
**2021**	35024
**2022**	28532

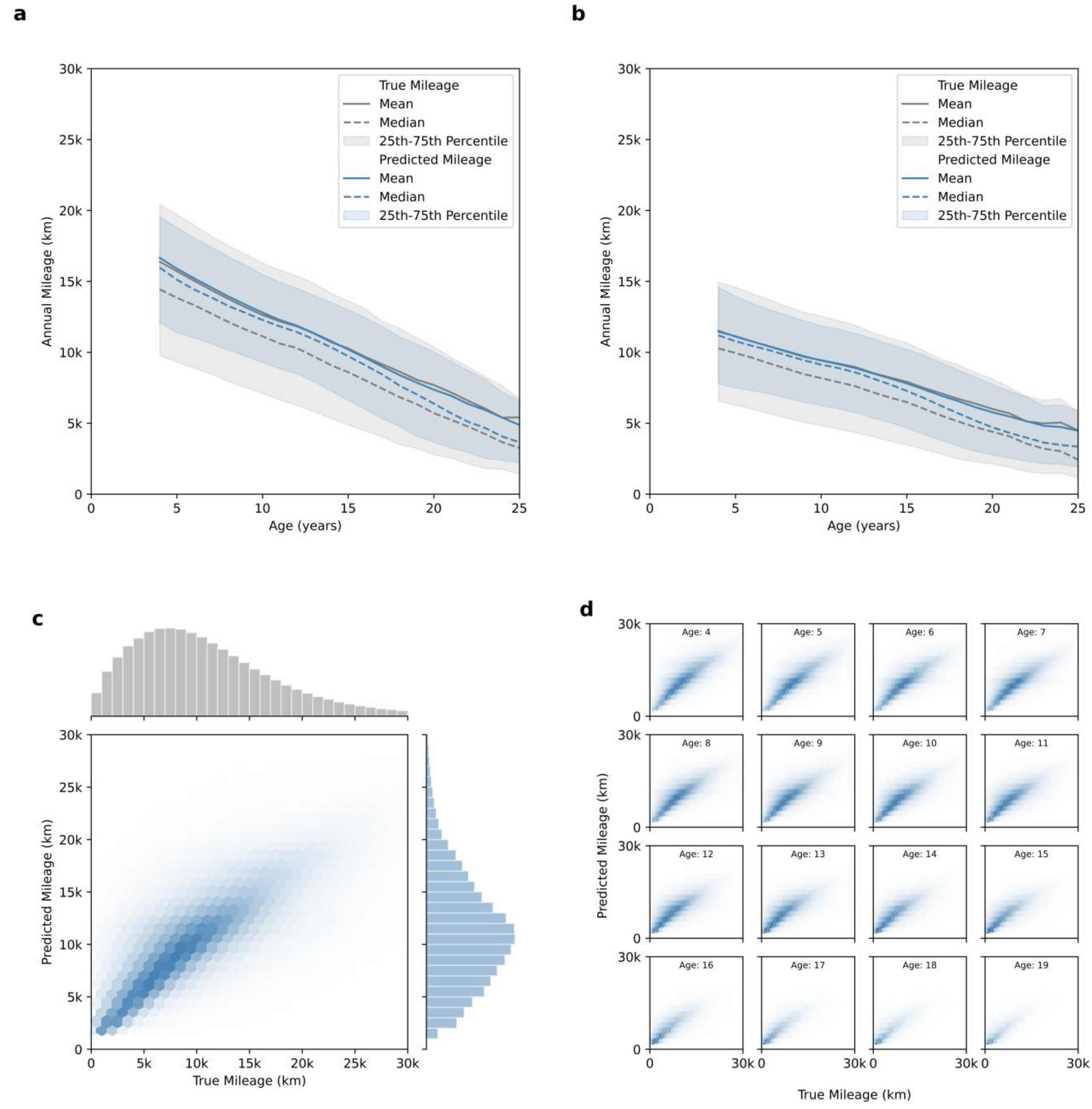

Supplementary Figure 3. Model validation of predicted annual vehicle mileage. The median and mean true and predicted annual mileage by vehicle age, given for diesel (a) and petrol (b) with interquartile ranges given with shaded areas; c gives a heatmap for true and predicted annual mileage with histograms for each above and to the right of the plot, respectively, using the same grey and blue colours for true and predicted values, respectively; d provides a repeat of the heatmap in c but for each vehicle age between 4 and 19.

Supplementary Table 6. Model validation of mileage and survival prediction. AUC-ROC: Area under the receiver-operating characteristic curve.

**Target Variable**	**Metric**	**Value**
**Mileage**	Root Mean Squared Error	5,828 km
**Mileage**	Mean Absolute Error	3,663 km
**Mileage**	Median Absolute Error	2,308 km
**Mileage**	R-Squared	0.49
**Survival**	Accuracy	0.88
**Survival**	Precision	0.41
**Survival**	Recall	0.36
**Survival**	F1 Score	0.38
**Survival**	AUC-ROC	0.83

Supplementary Table 7. Model validation of mileage and survival prediction for BEV and HEV leave-last-test-out evaluation. Abbreviations: R^2 (R-Squared): coefficient of determination; F1 Score: harmonic mean of precision and recall; AUC-ROC: Area under the receiver-operating characteristic curve.

**Target Variable**	**Fuel Type**	**Metric**	**Value**
**Mileage**	BEV	Root Mean Squared Error	4433 km
**Mileage**	BEV	Mean Absolute Error	2794 km
**Mileage**	BEV	Median Absolute Error	1773 km
**Mileage**	BEV	R-Squared	0.48
**Mileage**	HEV	Root Mean Squared Error	5944 km
**Mileage**	HEV	Mean Absolute Error	3398 km
**Mileage**	HEV	Median Absolute Error	2794 km
**Mileage**	HEV	R-Squared	0.58
**Survival**	BEV	Accuracy	0.94
**Survival**	BEV	Precision	0.24
**Survival**	BEV	Recall	0.52
**Survival**	BEV	F1 Score	0.33
**Survival**	BEV	AUC-ROC	0.71
**Survival**	HEV	Accuracy	0.93
**Survival**	HEV	Precision	0.40
**Survival**	HEV	Recall	0.38
**Survival**	HEV	F1 Score	0.39
**Survival**	HEV	AUC-ROC	0.77

Supplementary Table 8. Adjustment factors for type-approval fuel consumption to real world. Abbreviations: New European Driving Cycle (NEDC), Worldwide Harmonised Light Vehicle Test Procedure (WLTP), Hybrid Electric Vehicle (HEV).

**Fuel Type**	**Cylinder capacity**	**NEDC to Real World**	**WLTP to Real World**
**Petrol**	< 1.4	1.48	1.19
**Petrol**	1.4 – 2.0	1.48	1.29
**Petrol**	> 2.0	1.16	1.08
**Diesel**	< 1.4	1.37	1.09
**Diesel**	1.4 – 2.0	1.37	1.13
**Diesel**	> 2.0	1.37	1.20
**HEV**	< 1.4	1.46	1.07
**HEV**	1.4 – 2.0	1.45	1.11
**HEV**	> 2.0	1.45	1.19

Supplementary References

1. Mehlig, D., Staffell, I., Stettler, M. & ApSimon, H. Accelerating electric vehicle uptake favours greenhouse gas over air pollutant emissions. *Transp. Res. Part Transp. Environ.* **124**, 103954 (2023).
2. Wakeling, D., Wong, J., Karagianni, E. & Murrells, T. *Improvements to Road Transport Methodologies - Fleet Turnover and COPERT*. http://naei.energysecurity.gov.uk/reports/improvements-road-transport-methodologies-fleet-turnover-and-copert (2025).
3. Department for Transport. Vehicle licensing statistics data tables. (2024).
4. Nguyen-Tien, V., Zhang, C., Strobl, E. & Elliott, R. J. R. The closing longevity gap between battery electric vehicles and internal combustion vehicles in Great Britain. *Nat. Energy* 1–11 (2025) doi:10.1038/s41560-024-01698-1.
5. Paszke, A. *et al.* Pytorch: An imperative style, high-performance deep learning library. *Adv. Neural Inf. Process. Syst.* **32**, (2019).
