# Supplementary material for: Real-world mileage and survival of electric vehicles in Britain and implications for lifecycle emissions
Source: Nat Commun. 2026 Sep 21;17:10989. doi: 10.1038/s41467-026-77892-4 (PMC13683361; doi:10.1038/s41467-026-77892-4)
Supplement: Supplementary file 3 [file 41467_2026_77892_MOESM3_ESM.pdf]

nature portfolio

Peer Review File

Real-world mileage and survival of electric vehicles in Britain and implications for lifecycle emissions

Corresponding Author: Dr Daniel Mehlig

This file contains all reviewer reports in order by version, followed by all author rebuttals in order by version.

Version 0:

Reviewer comments:

Reviewer #1

(Remarks to the Author)
This study analyses evolution of EV lifetime and its impact on EV life cycle carbon footprint. It examines whether the increasing EV mileage will result in life cycle GHG benefits by combining statistical analysis and life cycle analysis. The data processing and survival rate analysis has been nicely done based on robust vehicle mileage and attribute data.

In the GHG calculations, however, several potentially critical issues are found in the selection of coefficients and parameters. It is uncertain whether the results (e.g., mid-sized EVs have lower emissions than small- or large EVs) will remain valid when they are updated with more realistic values. A sensitivity or uncertainty analysis of key parameters is necessary to improve the model and validate its key findings. Detailed comments with recommendations are below.

- There seems to be ambiguities and misunderstandings in estimating real-world fuel consumption values. First, in lines 395-98, the authors use the type-approval to real-world energy correction factors by Dornoff et al. However, the factors in the references do not cover EVs. For ICEVs, the references provide divergent year by year factors. It is not clear what factors the authors use for different powertrain types in the paper. In addition, there is a charging loss 10% assumed when calculating emissions (line 440). While NEDC fuel economy values do not include charging losses, WLTP fuel economy values for BEV do account for charging losses. Therefore, it is a double counting. To enhance clarity, first, an annual profile of type-approval test cycles used in the data is needed. Ideally, clear presentations of correction values across vehicle types, test cycles, test years are necessary. Considering uncertain nature of such factors, I would recommend a simpler and more generic approach, for example, as used in an EC vehicle LCA report, (doi: 10.2834/91418), for ratios between NEDC, WLTP, and real-world emissions.

- In line 429, the BEV battery production emissions of 65 kg CO2e per kWh seems to be a lower bound of literature values, if it corresponds to NMC, the most common battery type. The low value in the literature is primarily related to the overestimation of battery energy density (thus low material usages) at 4-5 kg /kWh, in early versions of GREET, together with a clean supply chain assumption. I recommend using a more realistic default value, for example, as in an EC vehicle LCA report, (doi: 10.2834/91418). As the carbon footprint of batteries vary depending on supply chain location, battery chemistry, and model year, and so on, in the literature, I would recommend a scenario analysis or sensitivity/uncertainty analysis for a range of battery production emission values.

- In line 428, vehicle manufacturing emission value of 3.6 kg CO2e per kg seems too low. It should be ~5 kg CO2/kg, as in the EC vehicle LCA (doi: 10.2834/91418) and an ICCT report, https://theicct.org/sites/default/files/publications/Global-LCA-passenger-cars-jul2021_0.pdf

- HEVs have much higher annual mileage and cumulative mileage than other powertrain types (Fig. 2). But their survival rate over age is comparable to ICEVs (Fig 3). Does this indicate that HEVs are more durable than other powertrains? Why HEVs are driven more than ICEVs? Do the trends simply reflect the lack of stability in HEV data? In fact, the HEV number of data point is higher than BEV. Adding discussions on these questions would be helpful.

- In line 67, MOT test is needed third year onward, but in line 139, vehicles enter MOT at three years of age, which seems to indicate that MOT test is done fourth year onward (age=current year – model year).

(Remarks on code availability)

Reviewer #2

(Remarks to the Author)
The authors leverage UK vehicle fleet data to analyze use trends and estimate life cycle emissions, and they train a transformer neural network on the data to project future BEV use, retirement and emissions, estimating that BEVs purchased in 2024 will have lifetimes similar to conventional vehicles and produce fewer greenhouse gas emissions over that lifetime.

This study is not appropriate for Nature Communications, but with some work it could be appropriate for a technical journal.

Major issues:
1. The authors need to address what happens to vehicles after they exit the UK fleet. Many vehicles are sold to secondary markets in other countries where they continue to be used and continue to produce greenhouse gas emissions.

2. The authors use average grid intensity, which is an outdated and inaccurate way to estimate the emissions consequences of charging EVs. I suggest reading the U.S. National Academy of Sciences report on Current Methods for Life Cycle Assessment of Low Carbon Transportation Fuels for more detail. There have also been several studies out recently finding that EV charging emissions are lower than one might expect because of effects on infrastructure investment [1-5].

3. It is not at all clear why we should trust the output of the transformer, what degree of uncertainty is associated with the predictions, or what kinds of validation have been performed. In particular, while the transformer may sound like a modern approach, the authors end up using the following assumption, which appears to predict EV life more by assumption than data: “For future predictions, BEVs and HEVs were classed as either petrol or diesel depending on their lifetime mileage of their final real-world test with a cutoff midway between each fuel type ”

4. In general, the relationships observed are not causal, but the forecasting exercise treats them as though they are. The data used are for small, old battery technologies over a period with a sparse charging network. It is not clear how such data could be use to reliably predict how modern EVs with modern battery technology would be used with current infrastructure. Additionally, the authors note “Despite concerns about the additional emissions burden of manufacturing larger battery packs for long-range BEV models32, they are currently driven more intensively than smaller BEVs, confirming preliminary evidence from the USA,” but there is no information about whether owners drive long range BEVs farther because they are long range or whether long-range drivers adopt BEVs at higher rates. The authors examine the travel behavior of the specific types of people who opted in to buying specific BEVs and compare it to the travel behavior of the kinds of people who didn’t — this does not tell us how those who didn’t adopt BEVs early might change their behavior when they do adopt BEVs in the future. Because the analysis lacks causal inference, it is not suited to make forecasts of future behavior.

5. The authors demonstrate a limited understanding of EV batteries. For example “Recent analysis suggests that battery degradation, the reduction in stored energy capacity and thus driving range, reduces capacity by 1.8%-2.3% per year33,34 with lower degradation reported more recently with newer vehicle models28.” This depends heavily on the specific lithium ion chemistry and the charging profile. There are many studies about this, and battery chemistry and technology differs over time and across vehicle models in systematic ways.

Overall, the study asks an interesting question and uses some interesting real-world data, but it is far to back-of-the-envelope and too speculative to be appropriate for Nature Communications.

[1] Bhandarkar, R., Luo, Q., Dimanchev, E., & Jenkins, J. D. (2025). Are EVs cleaner than we think? Evaluating consequential greenhouse gas emissions from EV charging. Environmental Research Letters, 20(10), 104041.
[2] Chen, J., Craig, M. T., Michalek, J., Bruchon, M., & Vaishnav, P. (2025). Negative Electric Vehicle Emissions: Vehicle-to-Grid Can Incentivize Enough Wind and Solar Investment to Reverse EV Charging Emissions. Environmental Science & Technology
[3] Gagnon P, Cole W. Planning for the evolution of the electric grid with a long-run marginal emission rate. iScience. 2022 Feb 11;25(3):103915. doi: 10.1016/j.isci.2022.103915. PMID: 35243264; PMCID: PMC8873608.
[4] Hanig, L., Weis, A., Jaramillo, P., & Michalek, J. (2025). Driving the grid forward: How electric vehicle adoption changes power-sector emissions (uses long-run consequential estimates for EV charging). Proceedings of the National Academy of Sciences (PNAS).
[5] Holland, S. P., Mansur, E. T., & Yates, A. J. (2024). Decarbonization and Electrification in the Long Run. NBER working paper

(Remarks on code availability)

Reviewer #3

(Remarks to the Author)
The information presented in this article is very important and relevant as it closes a research gap. It provides information on annual mileage and survival rates of passenger cars separately by powertrain type. Especially the information for battery

electric vehicles (BEVs) presents new evidence showing that BEVs have a much shorter lifetime and lower annual mileage than internal combustion engine vehicles (ICEVs). BEVs registered today are used similar to ICEVs, having similar survival rates and lifetime mileages - a very important finding to inform life-cycle and total cost of ownership analyses.

Regarding content and methodology I have the following remarks:
• Hybrid electric vehicles (HEVs) are analyzed separately from gasoline and diesel ICEVs and the report explains that PHEVs are excluded from the analysis. Since mild-hybrid vehicles are growing fast in popularity but are present a fluent transition between HEVs and ICEVs, it would be important to understand if they are considered as HEVs or as ICEVs.
• While I understand the benefits of using a transformer neural network model for predicting future mileage and thereby the survival of vehicles in the fleet, it is also less transparent than using multivariate regression models. E.g. Suarez et al from the EU Joint Research Centre explain: "Compared to more complex machine learning approaches like random forest, MLR offers clearer insight into variable relationships and is better aligned with the needs of policy applications, where model transparency and reproducibility are critical." (https://doi.org/10.1016/j.scitotenv.2025.180454) Considering the high relevance of the reviewed article for policy making, it would be helpful to explain why this methodology was chosen.
• Figure 2: It is unclear how the bottom figure c) covers the years 2015-2023. Does it show the average over these years? And does it make sense showing an average value considering that the mileage changes a lot with registration year. Furthermore, why is the of figure c) only based on 2023 and the bottom on 2015-2023?
• LCA: It unclear how the LCA considers the second life of vehicle after they have left the fleet. Are all considered being scraped? And do you take into account the second life of batteries after the vehicles lifetime ended?
While the life-cycle emissions determined in this report for ICEVs and HEVs are in good agreement with ICCT findings, life-cycle emissions of BEVs are about 17% higher. It would be interesting to understand what leads to these differences (https://theicct.org/publication/electric-cars-life-cycle-analysis-emissions-europe-jul25/).
Charging losses for BEVs are assumed to be about 10%, however, the WLTP type-approval values already includes charging losses. Why were additional losses considered?
• Figure 3: How do you explain the sudden increase in BEV survival rate from 2015 to 2016?
• Line 170: I understood there's no data for 2023, so shouldn't this be 2022.
• Line 196/197 suggests that future survival mileage and age are determined by linear extrapolation based on the change from 2015 to 2023. However, it is not explained if the assumption of linear increase is justified based on the annual changes between 2015 and 2023.
• Line 205-207: How does the increase in cohort size at older age affect the derived survival curves? Can you estimate how much the survival rates are overestimated at max?
• The first sentence in line 270 seems to suggest that BEV lifetime increases the more a vehicle is used, while I understand the meaning should be that it increases for later registration years.
• Lines 358-363: I wonder if consumer behavior regarding relatively low-cost products like smartphones or computers can be transferred directly to high-price products like cars. Can you explain why think this is justified? Or, if it's more that the behavior observed on BEVs is the same as for other products, I would rephrase to explain this more clearly.
• The report provides explanation on why early BEVs were driven less than newer BEVs. It would be helpful to better explain the consumer-relevant differences between these early BEVs and current models (e.g. range, number of passengers, payload, charging speed).
• In the discussion, it seems that in a few occasions words are missing in some sentences.
• Line 470-473: I find this sentence very diffcult to understand. Consider rephrasing.

(Remarks on code availability)

Version 1:

Reviewer comments:

Reviewer #1

(Remarks to the Author)
Although the authors mostly addressed the previous comments, the discussion on the mileage and survival rate in particular for HEVs remains unconvincing although it is not the main focus of this study. Technical factors such as durability of specific model and fuel economy differences may not fully account for those mileage and survival data across powertrains. For example, the higher lifetime mileage of earlier HEVs may not be attributable to superior durability as their survival rate against vehicle age is comparable to gasoline vehicles. Furthermore, longer vehicle longevity alone may not explain the higher annual mileage. As a potential explanation, the authors may incorporate a discussion regarding the impact of policies in the UK. For example, the annual high mileage of full HEVs may have been partially driven by low-emission vehicle incentives such as parking perks or access to low emission zones. Likewise, incentives for BEVs and penalties for ICEVs may have contributed to their mileage profiles. These factors are especially relevant for high mileage applications such as commercial fleets, taxi cabs or ride-sharing vehicles. It would be also good to add a discussion on use patterns and mileages if the data allows such granularity.

(Remarks on code availability)

Reviewer #2

(Remarks to the Author)
I appreciate the caveats that the authors added to the revision to address my comments. In my view, these caveats are helpful but are not enough to address the fundamental issue and make this a Nature Communications-level contribution.

The fundamental issue is that the paper is posed as being able to address causal questions. This begins at the title: "Improving electric vehicle mileage and lifetime reinforces lifecycle emission benefits." Making this claim requires knowing what the consequences of improving electric vehicle mileage and lifetime are. This requires consequential analysis.

1) Attributional LCA is not the right tool to answer this question because it does not assess the effects of changes, such as changes in EV mileage and lifetime.
2) The effect of vehicle mileage and lifetime on lifecycle emission benefits depends on what happens to the vehicle and battery after its first life. It is understandable that the researchers lack data on what happens to vehicles and batteries after first life, but assuming zero additional life and zero displacement is not a neutral assumption.

This is not only a title wording issue. The authors train a transformer neural network to make projections and make claims about emissions that current BEVs will produce over their lifetimes. Making projections requires understanding causal relationships in the past data, rather than just extrapolating trends, because the mechanisms that caused correlations in early years (e.g.: lower range BEVs tended to be adopted by early adopters, low-distance drivers, multi-vehicle households, single family homes) structurally change in later years (higher range BEVs tended to be adopted by later adopters, longer-distance drivers, single vehicle households, multi-unit dwellings). And claims about emissions that BEVs "will produce" require modeling the difference in emissions between scenarios with BEVs to those without BEVs to estimate the change in emissions produced by adding BEVs.

The bottom line is that if the claims are causal (effects of changing EV mileage and lifetime), the analysis is not sufficient to support the claims, and if the claims are not causal (increasing EV mileage and lifetime associated with lower GHGs), then claims rewritten to reflect this would not be sufficiently novel or impactful for a broad interest journal like Nature Communications, in my view.

Taking each of my prior comments in turn:

1) Secondary markets: It is understandable that the researchers lack data on what happens to vehicles and batteries after they leave the UK vehicle fleet. The added paragraph is helpful, but the real task is to understand whether these unknowns have the potential to flip the conclusions. We would expect paths after first life to differ systematically between petrol/diesel vehicles and EVs – petrol/diesel vehicles might last longer and have more second life opportunities in countries with existing petrol infrastructure and limited charging infrastructure, while EV may be scrapped earlier and batteries may be recycled, especially in Europe with policy on recycled content. In either case, used vehicles or batteries may partly displace new production and may partly be additive. One way the authors could address this in the face of uncertainty is to flip the question and ask how different EV end of life pathways would need to be in order to change the conclusion of the paper.

2) Grid intensity: The use of attributional LCA and ISO 14040/14044 is not suitable for assessing the effect of changes, including the effect of improving EV mileage and lifetime identified in the paper's title. This is what consequential LCA is for.

3) Transformer predictions: The SI compares actual and predicted annual vehicle mileage across the fleet after the BEVs and HEVs have been reclassified as petrol or diesel vehicles. There are a few issues: (1) the predictions are systematically biased (e.g.: in Figure S3c the colored area is consistently above the 1:1 line for low mileage vehicles and consistently below the 1:1 line for high mileage vehicles); (2) it is not clear exactly what test is performed, but it looks like these are a within-sample fit statistics when what is needed is out-of-sample prediction error, such as cross validation; and (3) for the application, it is not enough that the model can predict the bulk fleet after the BEVs and HEVs have been reclassified as petrol and diesel vehicles; the claims are about future BEV performance specifically, so validation needs to be performed on predicting BEV mileage, lifetime, scrap rates, etc.

4) Causality: As noted above, the paper's framing is causal, despite the added caveats. It asks a causal question: how increases to EV mileage and lifetimes change lifecycle emissions benefits.

5) Batteries: Changes in batteries between BEVs sold in 2012 and 2023 include far more than just range. Battery chemistry, coatings, microstructure, thermal management, controls, cell format, production improvements, and other factors have all changed over this period, and all of these things affect battery life. For LFP cells, summarizing use via equivalent full cycles is reasonable, but for NMC degradation depends too heavily on other factors, such as depth of discharge, c-rate, temperature, etc. to be summarized this way, and larger packs will differ in depth of discharge, heat generation, etc. relative to smaller packs over the same drive cycle.

Overall, I think there is some very interesting data and analysis in this study, and I think it could do well in a transportation journal with some revision to address the comments above. I just think that the big-picture claims that can be supported by this analysis are too caveated and limited to be a good match for a broad-interest journal.

(Remarks on code availability)

Reviewer #3

(Remarks to the Author)
The information presented in this paper is very important and relevant as it closes a research gap. It provides information on annual mileage and survival rates of passenger cars separately by powertrain type. Especially the information for battery electric vehicles (BEVs) presents new evidence showing that older BEVs have a much shorter lifetime and lower annual mileage than internal combustion engine vehicles (ICEVs), whereas BEVs registered today are used similar to ICEVs, having similar survival rates and lifetime mileages - a very important finding to inform life-cycle and total cost of ownership analyses.
The work is presented in a transparent and comprehensible manner. It is great that the author makes both the source data and code publicly available. This enables conducting the analysis for other countries or markets.

Thank you for addressing my comments thoroughly. After the 2nd review, I have only three comments remaining:
* The small dots in Figure 1a are almost not discernable and the ones in yellow are almost not visible. I suggest: using larger dots; grouping "United Kingdom" with the other countries/regions and not "Global"; potentially using a different symbol for global which would allow using a different scaling for the countries/regions than for the global symbol.
* On page 14, the high mileage of HEVs is discussed. In some countries, Toyota HEVs are often used for ride-hailing services (Taxis, UBER etc). This could explain the high annual mileages. Vehicles with high annual mileages often show superior durabilty as they are operated much less of their lifetime in cold start mode (cold coolant/cold oil) and are serviced regularly. Therefore, the mentioned longevity might at least partly owed the vehicle usage and not only a better build and design quality. If this is the case in the UK, it could be mentioned.
* The last sentence in the same paragraph refers to micro- and mild-HEVs. It is difficult to understand that this sentence provides the reason for the declinining annual mileage of newer HEVs. Consider rephrasing. And the sentence gives the impression that consumers opt intentionally for a micro- or mild-hybrid (MHEVs) instead of a full-hybrid. However, MHEVs replace more and more conventional combustion engine vehicles (ICEV) and consumers might choose an MHEV without being aware of the hybridization, assuming they buy an ICEV.

Jan Dornoff (The International Council on Clean Transportation)

(Remarks on code availability)

Version 2:

Reviewer comments:

Reviewer #1

(Remarks to the Author)
The authors thoroughly addressed my comments providing additional insights and analyses. I have no further comments.

(Remarks on code availability)

Reviewer #2

(Remarks to the Author)
I appreciate the efforts that the authors have put in to making their case, but in my view the arguments are not persuasive on the central question of causality.

The central focus of the study is how new data on UK vehicle travel patterns and survival rates affects attributional life cycle estimates that have previously relied on assumptions for these factors. This is a fine thing to study, but it does not answer the larger questions claimed.

The revised title is improved in that it no longer makes a causal claim about the effects of improved mileage and lifetime on emissions.

But the first sentence of the abstract returns to causal language: “Battery electric vehicles (BEVs) are predicted to reduce lifecycle greenhouse gas emissions compared to conventional vehicles.” The claim that BEVs “reduce” lifecycle greenhouse gas emissions compared to conventional vehicles is causal: implying that the action of replacing a conventional vehicle with a BEV will result in reduced net GHG emissions. The use of the verb “reduce” makes this a consequential claim, not an attributional one, and answering it requires assessing how emissions change (e.g.: “reduce”) when conventional vehicles are replaced with BEVs. A corrected attributional claim would read something more like the following: “Attributional lifecycle assessments of conventional and electric vehicles typically report lower GHG emissions estimates for electric vehicles than for gasoline vehicles.” These attributional assessments are notably *not* estimates of how much emissions are “reduced” when conventional vehicles are replaced with BEVs. Rather, they represent a particular accounting framework filled with a combination of empirical data and modeling choices, such as where to draw a geographic boundary in an interconnected power grid to determine which emissions to assign to EV charging load in a particular region. Attributional LCA involves

arbitrary modeling choices about how to assign process emissions to coproducts, and the power grid has the largest collection of coproducts of any system, so the distinction between attributional estimates and causal claims is especially sharp.

Similarly, the last sentence of the abstract claims that BEVs “may produce” 99 g CO2 per km over their lifetime and that this confirms “their climate change mitigation potential.” Again, this claim is causal: implying that owning and operating an EV may “produce” a particular amount of emissions (relative to not owning an operating an EV) and that these attributional estimates are relevant for estimating the “mitigation potential” of switching from conventional vehicles to BEVs. Estimating mitigation potential requires estimating how much emissions mitigation is possible (e.g.: by replacing conventional vehicles with BEVs). Attributional LCA does not provide estimates of this because it does not attempt to estimate how emissions would change if conventional vehicles were replaced with BEVs.

Overall, the authors conduct an attributional assessment, but they use language throughout suggesting that they incorrectly interpret those attributional estimates as though they are causal and consequential. My view is that cleaning up this language to avoid any causal or consequential claims would make the paper accurate, and the contribution would be valuable to other life cycle modelers who conduct attributional LCA and would like to understand how new data on travel patterns and survival might affect their estimates. But because the analysis does not answer the causal questions that it currently claims, I do not think it is well suited for a broader audience. It would be better situated in an LCA journal because its primary audience is other LCA researchers, not researchers outside the field or policymakers wanting to understand the emissions implications of transitioning to BEVs.

My recommendation remains that this paper should strictly avoid causal claims about how BEVs might “reduce” emissions relative to conventional vehicles or offer “mitigation potential”, since attributional LCA does not estimate causal effects like these. Rather, the paper estimates how new data about lifetime and travel patterns affect attributional LCA estimates of BEVs and conventional vehicles. The paper will have value in an LCA journal, but without legitimate causal claims relevant to emissions reductions and climate change mitigation potential, I am skeptical of its value to a broader audience such as that targeted by Nature Communications.

(Remarks on code availability)

Reviewer #3

(Remarks to the Author)
Thank you for addressing my comments. As mentioned before, this publication will close a knowledge gap and will be helpful for future research and policy work. I therefore recommend the publication of this report.
I would also like to incourage the authors to regularly publish updated numbers on lifetime mileage and survival curves, considering the fast paced developments observed for electric vehicles.

My remaining comments only address typos and reference errors:
* In line 387, there is a word missing after "their"
* The references to the supplementary information need to be checked. E.g. the main document refers to Table 6 in the supplement, but tables are labeled as e.g. Table S6. Furthermore, additional information was introduced in the supplement, so table numbering changed. This also led to two tables being labeled as S7.

(Remarks on code availability)

Version 3:

Reviewer comments:

Reviewer #2

(Remarks to the Author)
The authors have addressed my comments by rewording text across the manuscript to appropriately contextualize the results within attributional LCA and to avoid making causal claims. They also provided evidence of other studies in Nature Communications and Nature Sustainability that rely on attributional LCA in order to argue for broad relevance despite the absence of causal claims. These are all reasonable. My remaining request is that the authors include a sentence when discussing limitations of the study indicating that future work on causal and consequential analysis could leverage these results to help estimate emissions implications of corporate, consumer or policy actions, such as encouraging increased BEV adoption or influencing vehicle scrappage patterns.

(Remarks on code availability)

Open Access This Peer Review File is licensed under a Creative Commons Attribution 4.0 International License, which permits use, sharing, adaptation, distribution and reproduction in any medium or format, as long as you give appropriate credit to the original author(s) and the source, provide a link to the Creative Commons license, and indicate if changes were made.
In cases where reviewers are anonymous, credit should be given to 'Anonymous Referee' and the source.
The images or other third party material in this Peer Review File are included in the article's Creative Commons license, unless indicated otherwise in a credit line to the material. If material is not included in the article's Creative Commons license and your intended use is not permitted by statutory regulation or exceeds the permitted use, you will need to obtain permission directly from the copyright holder.
To view a copy of this license, visit https://creativecommons.org/licenses/by/4.0/

Response to Reviewers

We thank all three reviewers for their constructive comments that have helped to improve this manuscript. We agreed with the reviewers' recommendations and have incorporated them into the revised manuscript.

To summarise our main changes to the manuscript:

- We have revised our assumptions for emissions from manufacturing vehicles and batteries, which increases lifetime emissions for all vehicles, but leaves our overall conclusions unchanged.
- We have included a sensitivity study to understand the impact of our assumptions about battery manufacturing emissions. We find that both our main life cycle emission conclusions still hold: (i) first-generation BEVs, which did not last as long or were driven as much, still delivered lower lifecycle CO_{2e} emissions per kilometre than HEVs, petrol, and diesel cars; and (ii) projecting the mileage and lifetime of the current stock forward, BEVs still provide the by far the lowest lifetime CO_{2e} emissions per kilometre.
- We have clarified our assumptions about export of vehicles and use in second-life applications. These are excluded for simplicity, and because more complex treatment would be purely hypothetical without real world data. This is now properly communicated to readers, with thoughts on the impact these choices have on results.
- Since another year of MOT data have been released for tests taken in 2024, we updated our analysis to include this. This makes our the only paper we are aware of to use this new dataset, and increases our sample size and duration, thus making results more robust. We find the overall trends we report and illustrated in figures in the first submission are not changed.
- Finally, we revised the Discussion section to begin with the big picture implications of the paper and conclude with a forward-looking statement of how future research could further this work.

Below, we respond to each reviewer's comment individually, and quote changes to the manuscript in *italics*.

Response to Reviewer #1

This study analyses evolution of EV lifetime and its impact on EV life cycle carbon footprint. It examines whether the increasing EV mileage will result in life cycle GHG benefits by combining statistical analysis and life cycle analysis. The data processing and survival rate analysis has been nicely done based on robust vehicle mileage and attribute data.

> **Response:** Thank you for your kind words about the work and providing your comments.

In the GHG calculations, however, several potentially critical issues are found in the selection of coefficients and parameters. It is uncertain whether the results (e.g., mid-sized EVs have

lower emissions than small- or large EVs) will remain valid when they are updated with more realistic values. A sensitivity or uncertainty analysis of key parameters is necessary to improve the model and validate its key findings. Detailed comments with recommendations are below.

> **Response:** Thank you for raising these issues on our parameter selection. We have amended the parameters following the comments below. With these revisions we find larger lifecycle emissions for all vehicles, with BEVs seeing the largest increase but remaining the lower than HEVs, petrol and diesel vehicles. We also continue to find that mid-sized BEV provides the lowest emissions on a per kilometre basis out the three battery sizes considered, meaning the overall qualitative conclusions have not changed.

- There seems to be ambiguities and misunderstandings in estimating real-world fuel consumption values. First, in lines 395-98, the authors use the type-approval to real-world energy correction factors by Dornoff et al. However, the factors in the references do not cover EVs. For ICEVs, the references provide divergent year by year factors. It is not clear what factors the authors use for different powertrain types in the paper. In addition, there is a charging loss 10% assumed when calculating emissions (line 440). While NEDC fuel economy values do not include charging losses, WLTP fuel economy values for BEV do account for charging losses. Therefore, it is a double counting. To enhance clarity, first, an annual profile of type-approval test cycles used in the data is needed. Ideally, clear presentations of correction values across vehicle types, test cycles, test years are necessary. Considering uncertain nature of such factors, I would recommend a simpler and more generic approach, for example, as used in an EC vehicle LCA report, (doi: 10.2834/91418), for ratios between NEDC, WLTP, and real-world emissions.

> **Response:** Thank you for highlighting the shortcomings in our real-world corrections for fuel consumption. We have now adopted the approach you recommend from Hill et al 2020, (doi: 10.2834/91418), and added these values as a table to the Supplementary Material, Table S6.
>
> Regarding the real-world energy consumption of BEVs, the values are taken from the electric vehicle database that are not WLTP figures. We cover BEV models from 2012 to 2021 and so most of these registration years do not have WLTP energy consumption figures. The energy consumption values from the EV database are based upon an aggregation of driving conditions and temperature conditions for Europe and do not include charging losses, hence we add them.
>
> We have amended the section that covers BEV energy consumption in the methods to read:
>
> *"The third database gave vehicle attributes for BEVs, providing battery capacity in kWh and energy consumption in kWh per km[13]. The database provides energy consumption for real-world conditions for BEVs operating in European driving and weather conditions where upstream losses from charging were considered separately (see Lifecycle greenhouse gas emissions section below). We include BEVs models from 2012 to 2021, thus spanning periods where NEDC and WLTP drive-cycles were used to calculate energy consumption. The EV*

> *database uses these reported drive-cycle values to provide a consistent estimation of real-world BEV energy consumption across this period[63].*"

- In line 429, the BEV battery production emissions of 65 kg CO2e per kWh seems to be a lower bound of literature values, if it corresponds to NMC, the most common battery type. The low value in the literature is primarily related to the overestimation of battery energy density (thus low material usages) at 4-5 kg /kWh, in early versions of GREET, together with a clean supply chain assumption. I recommend using a more realistic default value, for example, as in an EC vehicle LCA report, (doi: 10.2834/91418). As the carbon footprint of batteries vary depending on supply chain location, battery chemistry, and model year, and so on, in the literature, I would recommend a scenario analysis or sensitivity/uncertainty analysis for a range of battery production emission values.

> **Response:** Thank you for pointing out the wider literature available on battery manufacturing emissions and explaining the bias in some literature values. We have amended our assumption to use 89 kg CO_{2e} per kWh as the central value base upon Hill et al 2020, (doi: 10.2834/91418). We now provide a sensitivity study for the upper and lower range provided, ranging from 78 to 101 kg CO_{2e} per kWh and have added this paragraph under the results section for lifecycle greenhouse gas emissions:
>
> "*Battery manufacturing emissions contribute a significant portion of BEV lifecycle emissions. With a mean battery capacity of 58 kWh for BEVs in our data, this equated to 5,200 kg of CO_{2e} or approximately 30 g CO_{2e} per km over the vehicle's lifetime. However, estimating these manufacturing emissions is sensitive to the assumed energy mix used to produce the batteries, with Hill et al.[36] reporting a range of 78 to 101 kg CO_{2e} per kWh. This uncertainty would mean that BEV manufacturing emissions could range by up to ±700 kg of CO_{2e} or approximately ±4 g CO_{2e} per km over the vehicle's lifetime.*"

- In line 428, vehicle manufacturing emission value of 3.6 kg CO2e per kg seems too low. It should be ~5 kg CO2/kg, as in the EC vehicle LCA (doi: 10.2834/91418) and an ICCT report, https://theicct.org/sites/default/files/publications/Global-LCA-passenger-cars-jul2021_0.pdf

> **Response:** Thank you for pointing us to additional data sources. We have updated our assumption to be 5.2 kg CO_{2e} per kg for ICEVs and 4.7 kg CO_{2e} per kg for BEVs based upon Bieker (2021).

- HEVs have much higher annual mileage and cumulative mileage than other powertrain types (Fig. 2). But their survival rate over age is comparable to ICEVs (Fig 3). Does this indicate that HEVs are more durable than other powertrains?

> **Response:** Thank you for raising this interesting discussion point on HEVs. We speculate that the better longevity of HEVs compared to petrol and diesel cars in terms of mileage, may be due to the manufacturers producing these vehicles. For example, in 2015 Toyota and Lexus were responsible for 87% of all new HEVs, but their share dropped to 30% in 2021. This manufacturer is known for long warranty period compared to competitors. The share of manufacturers producing HEVs is more diverse in 2021 and closer to the shares

> producing petrol and diesel cars. Therefore, the longevity of HEVs may tend towards petrol and diesel vehicles in the longer term.
>
> Please note we amend the manuscript with this and the three further points below in one new paragraph.

Why HEVs are driven more than ICEVs?

> **Response:** We cannot provide a causal answer, but we can speculate that could be due to lower real world fuel consumption and lower maintenance requirements of HEVs relative to petrol or diesel vehicles. Consumers might choose these vehicles if they have higher mileage demands to reduce annual running costs. This provides similar economic benefits as diesel vehicles and so may explain their similar mileage trends.
>
> This matches the explanation given by Kinsella (10.1016/j.rser.2023.113160) and in various online articles about HEV usage from The RAC, Carwow and Inshur.

Do the trends simply reflect the lack of stability in HEV data?

> **Response:** To summarise the above points and a similar point raised by Reviewer 3, we think there are a few reasons that could explain the trends in the HEV mileage and survival data. Firstly, the growth of mild hybrids could be contributing to lower mileage since these vehicles are not as fuel efficient as full hybrids. Secondly, as mentioned above, Toyota's share of the HEV market has fallen, and so the HEV data reflects the changing mix of manufacturers, who offer vehicles with different longevity and fuel consumption characteristics.

In fact, the HEV number of data point is higher than BEV. Adding discussions on these questions would be helpful.

> **Response:** Thank you for raising these interesting discussion points. We have added a paragraph in the discussion covering the above three responses:
>
> *"HEV lifetime mileage is also still changing. It appears to already exceed that of diesel vehicles at 230,000 km, but could be trending downwards. MOT data show that Toyota produced 87% of new HEVs in 2015, dropping to 30% by 2021 as more manufacturers entered the HEV market. The known longevity of Toyota vehicles[45] may explain the higher lifetime mileage of earlier HEVs, which now is trending towards the fleet mean for petrol and diesel as the mix of manufacturers diversifies. Likewise, the annual mileage of HEVs equalled diesel vehicles for earlier model years but is declining in more recent years. Both diesel and HEVs benefit from lower fuel consumption and are more economical for high mileage consumers. However, the rising share of micro-HEVs and mild-HEVs (which are less economical than full-HEVs[46,47]) may be more suitable for lower-mileage consumers."*

- In line 67, MOT test is needed third year onward, but in line 139, vehicles enter MOT at three years of age, which seems to indicate that MOT test is done fourth year onward (age=current year – model year).

Response: Thank you for highlighting the lack of clarity in our language. We have updated the text from line 139 to clearly state that the first test is at age three:

"Vehicles have their first MOT test at age 3, so we combine this…"

Response to Reviewer #2

The authors leverage UK vehicle fleet data to analyze use trends and estimate life cycle emissions, and they train a transformer neural network on the data to project future BEV use, retirement and emissions, estimating that BEVs purchased in 2024 will have lifetimes similar to conventional vehicles and produce fewer greenhouse gas emissions over that lifetime. This study is not appropriate for Nature Communications, but with some work it could be appropriate for a technical journal.

Major issues:

1. The authors need to address what happens to vehicles after they exit the UK fleet. Many vehicles are sold to secondary markets in other countries where they continue to be used and continue to produce greenhouse gas emissions.

Response: Thank you for highlighting this key assumption on vehicle exports. There is no granular data on what happens to exported vehicles (e.g. their miles driven or subsequent lifetime), so we do not include the export of vehicles to other markets. To understand what proportion of vehicles this assumption affects from our analysis, we observe that 15% of the vehicles we assume are scrapped are exported to another country based upon statistics from the ITF Used Vehicle Registration Database. We note that an important previous study by Nguyen-Tien et al. (2025) (https://doi.org/10.1038/s41560-024-01698-1) which used the same MOT data to estimate vehicle survival also acknowledged this limitation, and that current data do not permit exported vehicles to be studied meaningfully.

To address this, we have added a new paragraph in the Discussion to cover this point and to include findings from two recent LCAs that have covered this topic of underestimating vehicle lifetime:

"The MOT data covers vehicles while they are active in the fleet, so we assume vehicles exit through scrappage or export once they are no longer active. Public data do not identify which vehicles are sold internationally as second-hand exports. Using the ITF Used Vehicle Registration Database[49], we find that ~15% of vehicles exiting the fleet are exported. The MOT data therefore underestimate true vehicle lifetime and mileage. Recent LCAs estimate that exported vehicles see 20-33% additional lifetime mileage beyond domestic licensing statistics, leading to reductions in lifetime CO_{2e} of 7-22% for BEVs, 7-11% for HEVs and 4-8% for petrol and diesel vehicles[36,50]. Factoring this into our results would further increase the climate benefits of BEVs relative to conventional vehicles. We also exclude the emissions effects of second life applications for BEV batteries, which previous studies indicate could reduce BEV lifetime emissions by a further 1 g CO_{2e} per km[36]"

2. The authors use average grid intensity, which is an outdated and inaccurate way to estimate the emissions consequences of charging EVs. I suggest reading the U.S. National Academy of Sciences report on Current Methods for Life Cycle Assessment of Low Carbon Transportation Fuels for more detail. There have also been several studies out recently finding that EV charging emissions are lower than one might expect because of effects on infrastructure investment [1-5].

> **Response:** Thank you for raising the important choice regarding emission intensity calculation. We agree that there are strong alternative methods to using average emission factors for assessing the integration of EVs into electricity systems, and we had not sufficiently justified our choice.
>
> The choice of emission intensity calculation depends upon the aim of the study and the research question posed. It is widely accepted that consequential LCAs (those assessing the impact of decisions) should use short and long run marginal emission factors as you suggest. Our study is an attributional LCA of vehicles already in the British vehicle fleet, where we do not consider their impact on current or future decisions. We use the recommendations in ISO 14040/14044 to use the appropriate emission factor approach suitable for our research question. For our case as an attributional study, we use average emission factors. Likewise, each LCA we have cited in the text follow the same attributional approach which furthers our rationale for choosing this method of emission accounting.
>
> We have added to the text outlining this decision in the methods where we cover the emission intensity of electricity:
>
> *"Following the recommendations in ISO 14040/14044 to use the appropriate emission factor accounting method for the lifecycle analysis research aim, we used average emission intensity rather than a short- or long-run marginal emission intensity[32–34]. This decision was due to our study providing an attributional lifecycle analysis for vehicles already in the British vehicle fleet and not assessing consequences from decisions. By using yearly averages for electricity emissions intensity, we note that historical CO_{2e} emissions up to 2020 may be underestimated by up to 5% due to the current coincidence of real-world charging profiles and the diurnal pattern of electricity emission intensity[31]."*
>
> We also briefly note how other methods beyond average grid intensity could be used when we first introduce this aspect of the study in the Results section:
>
> *"We note that alternative ways to represent grid carbon intensity, such as short and long run marginal emissions, can yield different results than the average factors used here[31–34]"*

3. It is not at all clear why we should trust the output of the transformer, what degree of uncertainty is associated with the predictions, or what kinds of validation have been performed. In particular, while the transformer may sound like a modern approach, the authors end up using the following assumption, which appears to predict EV life more by assumption than data: "For future predictions, BEVs and HEVs were classed as either petrol or diesel depending on their lifetime mileage of their final real-world test with a cutoff midway between each fuel type "

> **Response:** Thank you for highlighting the necessary assessment of the model's accuracy and validity of applying it to EVs. Standard model validation metrics, such as R-Squared,

Root Mean Squared Error and further metrics are provided in Supplementary Material Figure S3 and Table 6 (this has been moved from Extended Data Figure 6 and Table 1). We have added a brief summary of these validation metrics to the manuscript when we first introduce the model.

Regarding the application of the model to estimate EV mileage and lifetime, the first half of our study demonstrates how the current generation of EVs are trending towards the same usage trends of diesel and petrol vehicles, and will match the usage and lifetime in the next few years. We therefore make a necessary assumption that we can use these petrol and diesel trends to simulate how EVs will be used over the remainder of their lifetimes – as to the best of our knowledge there is no more defensible approximation that can be made. The true lifetime and usage of current-generation BEVs cannot be known until the latter half of the 2030s, when we would expect half of the 2024 model-year vehicles to have exited the fleet; hence a proxy is necessary here.

We do not claim that our model can foresee how EVs will be used in the real world, but use it to provide an approximation based upon the current trends of the British vehicle fleet. We have added to the section outlining this assumption:

"*Based upon the observed trends in Figures 2 and 3, which demonstrate that BEV and HEV mileage and lifetimes are converging toward those of petrol and diesel vehicles, we assume that future EV usage can be approximated using established patterns from conventional vehicles. For example, high-mileage BEVs, often equipped with larger batteries, exhibit usage trends similar to diesel vehicles, while lower-mileage BEVs with smaller batteries align more closely with petrol vehicle usage patterns. This provides an estimation of BEV and HEV mileage and lifetime beyond the currently available MOT data and should not be interpreted as predicting real-world values. As the MOT data does not yet include the full lifetimes of BEVs and HEVs, the training data included only petrol and diesel vehicles. For projections, each BEV and HEV was classified as either petrol or diesel based on its cumulative mileage at the last observed MOT test, using a cutoff value midway between the typical mileages of petrol and diesel vehicles (detailed in the Supplementary Information). This classification approach is justified by the observed trends toward parity in BEV and HEV mileage and survival rates with those of diesel and petrol vehicles, as shown in Figures 2 and 3.*"

4. In general, the relationships observed are not causal, but the forecasting exercise treats them as though they are. The data used are for small, old battery technologies over a period with a sparse charging network. It is not clear how such data could be use to reliably predict how modern EVs with modern battery technology would be used with current infrastructure. Additionally, the authors note "Despite concerns about the additional emissions burden of manufacturing larger battery packs for long-range BEV models[32], they are currently driven more intensively than smaller BEVs, confirming preliminary evidence from the USA," but there is no information about whether owners drive long range BEVs farther because they are long range or whether long-range drivers adopt BEVs at higher rates. The authors examine the travel behavior of the specific types of people who opted in to buying specific BEVs and compare it to the travel behavior of the kinds of people who didn't — this does not tell us how those who didn't adopt BEVs early might change their behavior when they do

adopt BEVs in the future. Because the analysis lacks causal inference, it is not suited to make forecasts of future behavior.

> **Response:** Thank for raising these important issues. We agree we that our approach cannot account for causality in the results and we do not provide forecasts of consumer behaviour.
>
> First, we have clarified in the text your point about large battery BEVs:
>
> "*Despite concerns about the additional emissions burden of manufacturing larger battery packs for long-range BEVs*[39]*, these models are currently driven more intensively than smaller BEVs, confirming preliminary evidence from the USA*[11]*. These data cannot determine whether consumers choose large-battery BEVs for their longer range, or whether high-mileage drivers adopt BEVs at higher rates."*
>
> Regarding your key point for how consumers who didn't adopt BEVs early might change their behaviour when they do adopt BEVs in the future. It is important to note that our approach estimates how long current EVs in the fleet will last if they mirror current trends. At no point do we claim to explore what could happen with *future* purchasers of EVs, who as you say may drive them differently. We have added to the discussion to cover this point:
>
> "*However, as BEV adoption grows, usage trends we report may change as consumer behaviours evolve, and these behaviours cannot be forecast from this study alone.* "

5. The authors demonstrate a limited understanding of EV batteries. For example "Recent analysis suggests that battery degradation, the reduction in stored energy capacity and thus driving range, reduces capacity by 1.8%-2.3% per year[33,34] with lower degradation reported more recently with newer vehicle models[28]." This depends heavily on the specific lithium ion chemistry and the charging profile. There are many studies about this, and battery chemistry and technology differs over time and across vehicle models in systematic ways.

> **Response:** Thank you for your concern, we had not clearly communicated our intention here. We do not mean to convey that this is a comprehensive model of battery degradation. The sentence you picked up on was an input to a small piece of context, and is not used in the main analysis. We use these numbers to qualify how the already-low range of old BEVs would become even lower with degradation, which might help to explain why a large share had exited the fleet. We have reworded this, explicitly labelling it as a "*simple thought experiment*" and "*(ignoring differences between chemistries, operating profiles, etc.)*" so as to not give readers the same first impression you received.
>
> We have also used our dataset to provide some more context, finding that older BEV batteries were cycled more intensely (given that they are smaller). As full equivalent cycles correlates with capacity loss, this provides another piece of context.
>
> We have rewritten this paragraph as:
>
> *"Our results suggest that BEVs registered in 2022-2024 may equal the lifetime mileage of petrol cars if current trends continue. Both usage and lifetime trends might partly reflect increasing battery capacity. Larger batteries enable longer journeys, making newer BEVs more versatile and thus used more. Battery degradation reduces useable capacity over time,*

> *further reducing range. As a simple thought experiment: if capacity falls by 1.8%-2.3% per year[40,41] (ignoring differences between chemistries, operating profiles, etc.), BEVs from 2012 would have lost up to 25% by 2023, exceeding the typical 20% end-of-life threshold[42,43]. Combining the 2012 cohort's mean battery size (22 kWh) and energy consumption (5.9 km per kWh) gives an average range reduction from 130 km to 98 km, which might help explain why 45% of this cohort had exited the fleet by 2024. Smaller batteries are also cycled more intensively per kilometre driven, implying faster degradation[44]. The 2012 cohort had accumulated a mean of 700 equivalent full cycles by 2024, and by age 5 they reached 350 cycles, with only 74% remaining. In contrast, the 2019 cohort (with 60 kWh capacity and 350 km range) had accumulated only 190 cycles by the same age, and 93% remained in 2024."*

Overall, the study asks an interesting question and uses some interesting real-world data, but it is far to back-of-the-envelope and too speculative to be appropriate for Nature Communications.

> **Response:** Thank you for highlighting our study's key strengths: the use of real-world mileage and survival data for all EVs in Britain to inform future lifecycle assessments. We respectfully disagree that the study is at all "back-of-the-envelope", except for the short aside in the Discussion about why low battery size and range might have influenced lower lifetimes in older vehicles, picked up in your previous comment. The results section of the paper is robustly at or beyond the state of the art, as we explain in the manuscript, and was kindly noted by Reviewers 1 and 3.

Response to Reviewer #3

The information presented in this article is very important and relevant as it closes a research gap. It provides information on annual mileage and survival rates of passenger cars separately by powertrain type. Especially the information for battery electric vehicles (BEVs) presents new evidence showing that BEVs have a much shorter lifetime and lower annual mileage than internal combustion engine vehicles (ICEVs). BEVs registered today are used similar to ICEVs, having similar survival rates and lifetime mileages - a very important finding to inform life-cycle and total cost of ownership analyses.

> **Response:** Thank you for your assessment of our work and for providing your comments.

Regarding content and methodology I have the following remarks:

• Hybrid electric vehicles (HEVs) are analyzed separately from gasoline and diesel ICEVs and the report explains that PHEVs are excluded from the analysis. Since mild-hybrid vehicles are growing fast in popularity but are present a fluent transition between HEVs and ICEVs, it would be important to understand if they are considered as HEVs or as ICEVs.

> **Response:** Thank you for highlighting this nuance. In the UK, mild and full HEVs are both classed and recorded as HEVs in government statistics. We have added this detail to the section introducing the MOT data:
>
> *"Both mild and full hybrids are labelled as HEVs in UK statistics and vehicle databases[26]".*

> We now mention this as a potential contributor to the downward trend in mean HEV mileage in recent years, as the growing share of mild hybrids within the HEV category might pull the overall down:
>
> *"This decline may be explained by the growing share of mild hybrids within the HEV category for UK vehicles since mild hybrids generally have smaller battery sizes and higher fuel consumption, thus are more appropriate to lower mileage consumers[28]. However, additional research (such as user surveys) would be needed to validate this claim."*

• While I understand the benefits of using a transformer neural network model for predicting future mileage and thereby the survival of vehicles in the fleet, it is also less transparent than using multivariate regression models. E.g. Suarez et al from the EU Joint Research Centre explain: "Compared to more complex machine learning approaches like random forest, MLR offers clearer insight into variable relationships and is better aligned with the needs of policy applications, where model transparency and reproducibility are critical." (https://doi.org/10.1016/j.scitotenv.2025.180454) Considering the high relevance of the reviewed article for policy making, it would be helpful to explain why this methodology was chosen.

> **Response:** Thank you raising the key detail about the choice of statistical model. We agree with Suarez's views on the trade-off between accuracy and transparency for their target audience. We argue that the model we have chosen is more appropriate due to the structure of our data, the combined task of regression and classification prediction, and the target audience. We now present these points in a new Discussion paragraph, and clearly raise the comparative benefits of multivariate regression (citing Suarez), and the issue of transparency and reproducibility with transformer neural networks:
>
> *"Our approach applies transformer neural networks to vehicle test data, which is to our knowledge the first such usage. This model was chosen because MOT test data comprises sequences of varying lengths, which suit transformer architectures for sequential inputs, and because the task requires simultaneous regression (predicting vehicle mileage) and classification (identifying whether a test represents the vehicle's final record). The model could be improved in future by incorporating vehicle mass as a predictor of lifespan[10,20] or integrating fuel consumption with fuel prices and weather conditions to forecast annual mileage[23]. Specific MOT test failure criteria could be added as predictor variables[18], but were excluded here because they are often fuel-type specific and would reduce applicability to BEVs. Although more transparent approaches, such as multivariate linear regression, offer interpretable insights into relationships that are useful for policy applications[48], our transformer model prioritises predictive accuracy for projecting vehicle usage and lifetimes. The model's code is made publicly available to enable replication studies. "*

• Figure 2: It is unclear how the bottom figure c) covers the years 2015-2023. Does it show the average over these years? And does it make sense showing an average value considering that the mileage changes a lot with registration year. Furthermore, why is the of figure c) only based on 2023 and the bottom on 2015-2023?

> **Response:** Thank you for pointing out these inconsistencies in Figure 2 – it was meant to only show 2023. We have amended panels c and f to use only use the latest year of data (which is now updated to 2024).

• LCA: It unclear how the LCA considers the second life of vehicle after they have left the fleet. Are all considered being scraped?

> **Response:** Thank you for highlighting this key assumption we did not clarify. We are not aware of any data that could be used to extend our analysis for vehicles once they leave Britain. Therefore, we cannot extend our methods to include additional longevity for these vehicles. We have added a new paragraph in the Discussion to cover this point and to include findings from two recent LCAs that have covered this topic of underestimating vehicle lifetime and mileage:
>
> *"The MOT data covers vehicles while they are active in the fleet, so we assume vehicles exit through scrappage or export once they are no longer active. Public data do not identify which vehicles are sold internationally as second-hand exports. Using the ITF Used Vehicle Registration Database[49], we find that ~15% of vehicles exiting the fleet are exported. The MOT data therefore underestimate true vehicle lifetime and mileage. Recent LCAs estimate that exported vehicles see 20-33% additional lifetime mileage beyond domestic licensing statistics, leading to reductions in lifetime CO_{2e} of 7-22% for BEVs, 7-11% for HEVs and 4-8% for petrol and diesel vehicles[36,50]. Factoring this into our results would further increase the climate benefits of BEVs relative to conventional vehicles. We also exclude the emissions effects of second life applications for BEV batteries, which previous studies indicate could reduce BEV lifetime emissions by a further 1 g CO_{2e} per km[36]."*

And do you take into account the second life of batteries after the vehicles lifetime ended?

> **Response:** Thank you for highlighting this key assumption. As above, to keep our focus on what can be well quantified with our dataset, we do not take into account any second life of batteries. We now make this clear to readers in the Discussion, and give them some context on how much it could affect our results:
>
> "[We] exclude the emissions effects of second life applications for BEV batteries, which previous studies indicate could reduce BEV lifetime emissions by a further 1 g CO_{2e} per km[36]."

While the life-cycle emissions determined in this report for ICEVs and HEVs are in good agreement with ICCT findings, life-cycle emissions of BEVs are about 17% higher. It would be interesting to understand what leads to these differences (https://theicct.org/publication/electric-cars-life-cycle-analysis-emissions-europe-jul25/).

> **Response:** Thank you for suggesting we compare our results to other key results. We find that our lifetime emission for BEVs was 99 g CO_{2e} per km. The ICCT found lifetime BEV emissions of 85 g CO_{2e} per km for a UK 2021 model. We associate most of the discrepancy to battery manufacturing emissions where we use 89 kg CO_{2e} per kWh vs their 65 kg CO_{2e}

> per kWh. Hill et al 2020 report 100 g CO_{2e} per km for UK BEVs, which is close to our result. We have added to the text covering this difference:
>
> *"Compared to recent studies using UK specific electricity generation emission intensities, we find our lifetime emissions for BEVs of 99 g CO_{2e} per kilometre is close to Hill et al[36] at 100 g CO_{2e} per kilometre but above Bieker[3] who found 85 g CO_{2e} per kilometre. This difference of 17% is explained primarily due to differences in embodied emissions from battery manufacturer where we assume a higher emission intensity of 89 kg of CO_{2e} per kWh36 (representative of the vehicles manufactured for the UK market between 2012 and 2021) compared to their 65 kg per kWh."*

Charging losses for BEVs are assumed to be about 10%, however, the WLTP type-approval values already includes charging losses. Why were additional losses considered?

> **Response:** Thank you for highlighting that we have not described our methods in enough detail. The energy consumption values from the electric vehicle database are not WLTP figures, they are based upon an aggregation of driving conditions and temperature conditions for Europe and do not include charging losses. We have amended the section that covers BEV energy consumption to:
>
> *"The third database gave vehicle attributes for BEVs, providing battery capacity in kWh and energy consumption in kWh per km[13]. The database provides energy consumption for real-world conditions for BEVs operating in European driving and weather conditions where upstream losses from charging were considered separately (see Lifecycle greenhouse gas emissions section below). We include BEVs models from 2012 to 2021, thus spanning periods where NEDC and WLTP drive-cycles were used to calculate energy consumption. The EV database uses these reported drive-cycle values to provide a consistent estimation of real-world BEV energy consumption across this period[63]."*

- Figure 3: How do you explain the sudden increase in BEV survival rate from 2015 to 2016?

> **Response:** Thank you for identifying this trend in the BEV survival data. We also see sudden increases between 2012 and 2013 and between 2013 and 2014. These increases are replicated in the survival curves derived from the alternative vehicle licensing data we use and so we assume these jumps do reflect changes in real world survival. The datasets alone cannot provide an explanation for these changes. We have noted this trend in the results section:
>
> *"The increase in BEV lifetime over time has not been consistent across model years. Subsequent model years have exhibited a step change in survival rates, as shown between BEV models from 2015 and 2016 in Figure 2a. We find this phenomenon is repeated for the survival curves derived from both data sets (see Supplementary Information Figure S1)"*

- Line 170: I understood there's no data for 2023, so shouldn't this be 2022.

> **Response:** Thank you for highlighting we were not clear in the text. With our updated MOT data including tests taken in 2024, we report results on vehicle mileage up to and including 2024 and vehicle survival up to and including 2022. This lower year for survival is due to the need to have a two year "buffer period" (from Nguyen-Tien et al., 2025) that

> enables us to determine if vehicles have left the fleet if they have no MOT records in the past two years. To make this clear we have added to the Vehicle survival section in the Results:
>
> *"We use the criteria from Nguyen-Tien et al.[10] to determine if a vehicle has exited the fleet which allows us estimate vehicle survival up until tests taken in 2022."*

• Line 196/197 suggests that future survival mileage and age are determined by linear extrapolation based on the change from 2015 to 2023. However, it is not explained if the assumption of linear increase is justified based on the annual changes between 2015 and 2023.

> **Response:** Thank you for highlighting this ambiguity. We have updated this extrapolation and applied a new method for calculating vehicle survival (please see our response to your comment below). To show how this growth has been linear, we have added a new figure to the Supplementary Information (Figure S2) showing the growth in BEV lifetime since 2016 with a clear linear trend. We have amended the text to cover this:
>
> *"Since 2016, BEV survival age has linearly increased by 0.6 years per year (see Supplementary Information Figure S2), reaching 11.2 years by 2022. If this linear trend continues, BEVs will match diesel vehicle lifetime of 13.9 years by 2027. Since vehicles enter the national fleet data at three years of age, limiting the latest registration year to 2021, this implies BEVs sold from 2024 onwards are likely to have comparable lifetime to petrol and diesel vehicles. The increase in BEV lifetime over time has not been consistent across model years. Subsequent model years have exhibited a step change in survival rates, as shown between BEV models from 2015 and 2016 in Figure 2a. We find this phenomenon is repeated for the survival curves derived from both data sets (see Supplementary Information Figure S1), but has not affected the recent consistent increase in survival from 2016."*

• Line 205-207: How does the increase in cohort size at older age affect the derived survival curves? Can you estimate how much the survival rates are overestimated at max?

> **Response:** Thank you for highlighting how we should discuss this increase in cohort size. Our previous method tracked the total number of vehicles in each cohort, which led to some year on year increases as vehicles age due to some vehicles missing an MOT test in a calendar year. In light of this shortcoming, we have revised the methods to now calculate survival based upon the number of vehicles that exit the fleet each year using the approach from Nguyen-Tien et al. (2025) to determine if a vehicle has left the fleet (10.1038/s41560-024-01698-1). This new method creates a monotonically decreasing survival curve so there are no instances of the survival curve now increasing at older ages.
>
> Please note that the first three years of survival come from the alternative UK government vehicle licensing statistics that track the total number of vehicles in each cohort, and so do still have this issue of increasing survival at older ages up to age four. We have changed the paragraph that previously noted this effect to now read:
>
> *"When estimating vehicle survival rates in the first three years using the Vehicle Licensing Statistics dataset[17], we occasionally observe small increases in the surviving cohort size at older ages. For example, Figure 3a shows that 2014 and 2015 model year BEVs exhibit an*

> *apparent ~2% rise in survival between age two and age three. This counter-intuitive increase occurs because the underlying number of licensed vehicles from these model years grew slightly in the licensing statistics. The licensing statistics only count vehicles that are currently taxed and on the road; they exclude vehicles that are untaxed, such as those held in dealerships on trade plates or temporarily legally declared off the road. When such vehicles are subsequently taxed and re-enter the licensed fleet, they reappear in the licensed total, creating the appearance of improved survival for that cohort, but these effects cannot be investigated separately using the publicly available data."*

• The first sentence in line 270 seems to suggest that BEV lifetime increases the more a vehicle is used, while I understand the meaning should be that it increases for later registration years.

> **Response:** Thank you for identifying this mistake in our language. We have removed this sentence from the text since other reviewer comments required this section to be changed.

• Lines 358-363: I wonder if consumer behavior regarding relatively low-cost products like smartphones or computers can be transferred directly to high-price products like cars. Can you explain why think this is justified? Or, if it's more that the behavior observed on BEVs is the same as for other products, I would rephrase to explain this more clearly.

> **Response:** Thank you for highlighting this. We have revised this section to add evidence specific to BEVs:
>
> *"For passenger cars, most consumers are delaying the purchase of an EV until technology and price meet expectations[59–61], reducing the value of first-generation BEVs that do not meet preferred performance criteria. Early BEVs might therefore have exited the fleet prematurely due to obsolescence compared to more advanced and affordable later models, rather than failure due to wear and tear."*

• The report provides explanation on why early BEVs were driven less than newer BEVs. It would be helpful to better explain the consumer-relevant differences between these early BEVs and current models (e.g. range, number of passengers, payload, charging speed).

> **Response:** Thank for suggesting we include more details about BEVs to hep explain these shifts. We have used more data from the EV database to aid the second paragraph of the discussion:
>
> *"Historically, BEVs were driven less intensively than conventional vehicles and did not survive as long in the British vehicle fleet. These trends have evolved rapidly as BEV technology has improved. Between 2012 and 2021, battery size increased from 22 kWh to 61 kWh, range increased from 130 km to 350 km, maximum charging speed increased from 46 kW to 104 kW, vehicle mass increased by 350 kg, and the most common body type shifted from hatchbacks (100%) to SUVs (43%). These combined technology shifts likely impact consumer attitudes[37]. Other factors beyond the vehicles themselves may also influence travel and purchase behaviour, such as the rapid increase in charging availability[38].*

We find that since 2017, new BEVs in Britain have been driven comparably to new conventional vehicles. Smaller-battery models equal the annual mileage of petrol, and larger-battery models equal that of diesel vehicles. By 2024, new BEVs and HEVs are approaching the usage of diesel cars, suggesting they are substituting demand for high-usage vehicles. As BEV adoption grows, usage trends we may change as consumer behaviours evolve, and these behaviours cannot be forecast from this study alone."

- In the discussion, it seems that in a few occasions words are missing in some sentences.

Response: Thank you for highlighting our mistakes in the text. We have reviewed and shortened the text and should have corrected any missing words.

- Line 470-473: I find this sentence very difficult to understand. Consider rephrasing.

Response: Thank you for catching this poorly worded section. We have rewritten this paragraph to be clear:

"Based upon the observed trends in Figures 2 and 3, which demonstrate that BEV and HEV mileage and lifetimes are converging toward those of petrol and diesel vehicles, we assume that future EV usage can be approximated using established patterns from conventional vehicles. For example, high-mileage BEVs, often equipped with larger batteries, exhibit usage trends similar to diesel vehicles, while lower-mileage BEVs with smaller batteries align more closely with petrol vehicle usage patterns. This provides an estimation of BEV and HEV mileage and lifetime beyond the currently available MOT data and should not be interpreted as predicting real-world values. As the MOT data does not yet include the full lifetimes of BEVs and HEVs, the training data included only petrol and diesel vehicles. For projections, each BEV and HEV was classified as either petrol or diesel based on its cumulative mileage at the last observed MOT test, using a cutoff value midway between the typical mileages of petrol and diesel vehicles (detailed in the Supplementary Information). This classification approach is justified by the observed trends toward parity in BEV and HEV mileage and survival rates with those of diesel and petrol vehicles, as shown in Figures 2 and 3.''

Response to Reviewers

We thank all three reviewers for their constructive comments that have helped to improve this manuscript. We agreed with recommendations from Reviewers 1 and 3 and have incorporated them into the revised manuscript.

We do not agree with many of the points made by Reviewer 2, and provide our response and evidence for our position below.

To summarise our main changes to the manuscript:

- To accommodate Reviewer 2's language concerns, we avoid any ambiguity over the methods being consequential by changing the title and some text in the abstract.
- To accommodate Reviewer 2's concern on the applicability of our model to BEVs and HEVs, we have provided additional model validation demonstrating that the model performs comparably well for BEVs and HEVs. We have added a new paragraph in the methods and a new table in the Supplementary Information.
- We have filtered the data for private hire vehicles and taxis to provide empirical data on the historical usage patterns of HEVs, replacing our earlier speculation. Two paragraphs have been revised that addresses comments by Reviewers 1 and 3 in the discussion and a new paragraph in the methods.
- Figure 1 has been revised to improve clarity that addresses Reviewer 3's comment.
- We added a sentence to show that it is not possible for secondary market usage to change our core finding, and revised a sentence about battery chemistry to reflect Reviewer 2's comments.

Below, we respond to each reviewer's comment individually, and quote changes to the manuscript in *italics*.

Response to Reviewer #1

Although the authors mostly addressed the previous comments, the discussion on the mileage and survival rate in particular for HEVs remains unconvincing although it is not the main focus of this study. Technical factors such as durability of specific model and fuel economy differences may not fully account for those mileage and survival data across powertrains. For example, the higher lifetime mileage of earlier HEVs may not be attributable to superior durability as their survival rate against vehicle age is comparable to gasoline vehicles. Furthermore, longer vehicle longevity alone may not explain the higher annual mileage. As a potential explanation, the authors may incorporate a discussion regarding the impact of policies in the UK. For example, the annual high mileage of full HEVs may have been partially driven by low-emission vehicle incentives such as parking perks or access to low emission zones. Likewise, incentives for BEVs and penalties for ICEVs may have contributed to their mileage profiles. These factors are especially relevant for high mileage

applications such as commercial fleets, taxi cabs or ride-sharing vehicles. It would be also good to add a discussion on use patterns and mileages if the data allows such granularity.

> **Response:** Thank you for highlighting the additional factors that likely impact HEV mileage. We agree that they could have influenced HEV annual mileage. We have added additional discussion on how policies, such as discounts on London's congestion zone, will have affected vehicle mileage for EVs.
>
> Also, based upon your suggestion, we have used the MOT test data to estimate the size of the private hire and taxi vehicle fleet, which provides new insight for this paragraph in the discussion:
>
> *"Annual mileage for HEVs was similar to diesel vehicles in earlier model years but is declining in more recent years. Both diesel and HEVs have lower fuel consumption than petrol vehicles, making them more economical for high mileage users, including commercial applications such as Private Hire Vehicles (PHVs) and taxis. The high mileage observed for HEVs might therefore be explained by a larger proportion of these vehicles being used in PHV and taxi services. Based on the MOT data, we estimate that in 2015, 13% of the HEV fleet aged 3–8 years were PHVs or taxis, far exceeding the 2-3% share for petrol and diesel vehicles. In 2015, the annual mileage of HEV PHVs and taxis were approximately twice the mean for diesel and HEVs (39,500 cf. 16,500 and 19,200 respectively), and three times the average for petrol (11,200). By 2024, the share of HEVs used as PHVs or taxis had declined to 5%, although these vehicles continued to record similarly increased annual mileage as in 2015.*
>
> *Policy incentives and consumer trends could have also influenced vehicle usage patterns. For example, exemptions from London's congestion charge for HEVs and BEVs[1] potentially encouraged higher mileage among these vehicles. In addition, the rising share of micro-HEVs and mild-HEVs, which provide smaller fuel-economy benefits than full hybrids[2,3], could have contributed to the decline in mean annual mileage for the overall HEV category (which includes micro, mild and full hybrids). Future work could investigate these influences, but the latter would require more granular data on vehicle classification."*
>
> We have added to the methods how we filtered the MOT data for these vehicles:
>
> *"Most vehicles operating as a private hire vehicle or taxi require an MOT test frequency of 6 months[4]. To produce an approximation of the population of private hire vehicles and taxis we filter the MOT data for vehicles that have had a test within 0.4 to 0.6 years. This filtering step will include a portion of vehicles that coincidentally have this time between MOT tests. Some local authorities have differing rules regarding MOT test frequency and so we cannot identify all taxis and private hire vehicles with this method. "*

Response to Reviewer #2

I appreciate the caveats that the authors added to the revision to address my comments. In my view, these caveats are helpful but are not enough to address the fundamental issue and make this a Nature Communications-level contribution.

> **Response:** Thank you for taking the time to review our study. We believe our work has been improved through responding to your comments.

The fundamental issue is that the paper is posed as being able to address causal questions. This begins at the title: "Improving electric vehicle mileage and lifetime reinforces lifecycle emission benefits." Making this claim requires knowing what the consequences of improving electric vehicle mileage and lifetime are. This requires consequential analysis.

> **Response**: Our title, "Improving electric vehicle mileage and lifetime reinforces lifecycle emission benefits", reflects one of our central empirical findings: that mileage and lifetime have improved over time for electric vehicles in Britain, and the downstream impact this has had on lifecycle emissions of vehicles that have already left the fleet. Reviewer 2 makes repeated claims that the language of our title necessitates a consequential analysis, citing *"the effect of changes, including the effect of improving EV mileage and lifetime identified in the paper's title."* This is a surface-level, language-based argument that misunderstands the reason we chose this title and the underlying attributional LCA methodology of our paper, as outlined above. To reduce the risk readers adopt the misconceptions of Reviewer 2, we are open to changing the title to: "Real-world mileage and survival of electric vehicles in Britain and implications for lifecycle emissions".
>
> To improve the clarity of our work we have revised the text in the abstract's use of *"we project that current BEVs will produce"* to "*we project that current BEVs may produce*". All other claims about future mileage, lifetime, and lifecycle emissions in the text are predicated on conditional statements of exiting trends, such as this unrevised sentence from the abstract: *"The annual mileage and lifetime of BEVs purchased in 2024 will equal those of conventional vehicles if recent trends continue"*.

1) Attributional LCA is not the right tool to answer this question because it does not assess the effects of changes, such as changes in EV mileage and lifetime.

> **Response:** The argument made here, and in the remainder of these comments, against the use of attributional LCA is a demonstrable misunderstanding of our study. We are using the same underlying methodology as every prior attributional LCA on electric vehicles for a reason.
>
> Our study's contribution is to add new data to provide the mileage and lifetime for existing vehicles in the British fleet and extrapolate these trends over the rest of their lifetime. We do not compare this new data with counterfactual scenarios of vehicle mileage and lifetime, and we do not assess how changes in mileage or vehicle lifetime translate into changes in lifecycle emissions, which is the argument put forth here.

> We observe the mileage and lifetime for all cars in Britian from 2005 to 2024. These are historical records of vehicles in Britian, and we use this to attribute the correct emissions for each vehicle based upon their unique mileage and lifetime. These are distinct records over time and are not counterfactual scenarios or theoretical changes. Regarding the projections of mileage and lifetime, we do not provide multiple counterfactual projections assessing decisions or changes. Rather, we use the unique history of each vehicle to project a single most likely future mileage and lifetime. This is fundamentally the same as previous attributional LCAs for BEVs. These prior attributional LCAs assume a single projection of annual mileage and lifetime for each vehicle and typically provide this for present day vehicles and vehicles a few years in the future. This is the same attributional LCA methodology that we use.
>
> It is wrong to claim that new empirical data on vehicle mileage and lifetime require a consequential analysis, on the grounds that our understanding of the technology has changed. By the same logic, any new information on electric vehicles would necessitate a consequential analysis; rendering attributional LCA obsolete as a research tool. This is demonstrably not how research in this field is conducted, as evidenced by the continued existence and publication of attributional LCAs providing updated understanding of electric vehicles[1-4].
>
> Attributional LCA is the primary and correct research tool for this purpose: using new empirical knowledge on electric vehicles to understand the emissions produced over their lifetimes. Every LCA cited in our manuscript uses an attributional approach for precisely this reason. There are, of course, many applications for consequential LCA, but our study is not one of them.

2) The effect of vehicle mileage and lifetime on lifecycle emission benefits depends on what happens to the vehicle and battery after its first life. It is understandable that the researchers lack data on what happens to vehicles and batteries after first life, but assuming zero additional life and zero displacement is not a neutral assumption.

> **Response:** Thank you for raising the importance of vehicle exports and battery second life. We provide discussion on this point based upon prior LCA studies evaluating the impact of vehicle exports and battery second life applications. These prior studies we reference used hypothetical assumptions because there is no data on this topic yet.
>
> In your later comment on this topic, we demonstrate how the second life cannot materially change the conclusions of our work; hence it is something suitable for future work rather than a necessity for our paper to consider. Devoting more attention to this when we can only speculate would be a distraction from the main analysis which we can quantify properly. Hence we do not speculate.

This is not only a title wording issue. The authors train a transformer neural network to make projections and make claims about emissions that current BEVs will produce over their lifetimes. Making projections requires understanding causal relationships in the past data, rather than just extrapolating trends, because the mechanisms that caused correlations in early years (e.g.: lower range BEVs tended to be adopted by early adopters, low-distance

drivers, multi-vehicle households, single family homes) structurally change in later years (higher range BEVs tended to be adopted by later adopters, longer-distance drivers, single vehicle households, multi-unit dwellings). And claims about emissions that BEVs "will produce" require modeling the difference in emissions between scenarios with BEVs to those without BEVs to estimate the change in emissions produced by adding BEVs.

> **Response:** We reject the fundamental proposition in this argument: to make projections requires statistical identification of causal relationships. This would potentially help to produce more accurate and robust projections, but is demonstrably not standard practice or a marker of novelty and impact. A recent and highly regarded study by Nguyen-Tien, et al.[5] in Nature Energy, used the same dataset we have used to project vehicle lifetime into the future. To do this they assumed BEVs would follow the same structural relationships of diesel and petrol vehicles. Causality was not considered in their study and is not mentioned in their discussion. We build on their work by adding mileage, correcting shortcomings in their survival methods, providing an alternative projection methodology using a transformer neural network, and finally apply this new understanding in an attributional LCA. It is simply incorrect to state this work needs to specifically provide statistical causation for our projections of vehicle mileage or lifetime.
>
> Moreover, the above comments by the reviewer demonstrate a further misunderstanding of our study. The argument that our study "require modelling the difference in emissions between scenarios with BEVs to those without BEVs to estimate the change in emissions produced by adding BEVs", demonstrates misunderstanding as we are not "adding BEVs". The BEVs in our study already exist and entered the British fleet between 2012 and 2021. We are not accessing decisions to add or remove BEVs and the consequential impact upon emissions, which is what this comment implies.

The bottom line is that if the claims are causal (effects of changing EV mileage and lifetime), the analysis is not sufficient to support the claims, and if the claims are not causal (increasing EV mileage and lifetime associated with lower GHGs), then claims rewritten to reflect this would not be sufficiently novel or impactful for a broad interest journal like Nature Communications, in my view.

> **Response:** This opinion on journal suitability provides the following "bottom line": that if our claims are causal, the analysis is insufficient to support them – and if our claims are associational, they are not sufficiently novel or impactful for Nature Communications. We reject both framings
>
> We have thoroughly refuted the claim that our analysis is insufficient in our earlier comments.
>
> On the question of novelty: our paper's primary contribution is to provide the first national-scale, real-world dataset on both battery electric vehicle (BEV) annual mileage and survival rates, and the application of these data to calculate the historical lifecycle emissions of vehicles currently in the British fleet. All prior LCA studies of electric vehicles have relied on assumed or theoretical values for mileage and lifetime, a widely acknowledged blind spot in the field. We further apply a transformer neural network to extrapolate empirically observed trends forward, enabling lifecycle emission projections

> grounded in real-world behaviour rather than simplified assumptions. To our knowledge, this is the first time that both real-world mileage and lifetime have been calculated for BEVs at national scale, and the first time a transformer neural network has been applied in this field. This constitutes a substantive empirical and methodological contribution that stands independently of any causal framing.

Taking each of my prior comments in turn:

1) Secondary markets: It is understandable that the researchers lack data on what happens to vehicles and batteries after they leave the UK vehicle fleet. The added paragraph is helpful, but the real task is to understand whether these unknowns have the potential to flip the conclusions. We would expect paths after first life to differ systematically between petrol/diesel vehicles and EVs – petrol/diesel vehicles might last longer and have more second life opportunities in countries with existing petrol infrastructure and limited charging infrastructure, while EV may be scrapped earlier and batteries may be recycled, especially in Europe with policy on recycled content. In either case, used vehicles or batteries may partly displace new production and may partly be additive. One way the authors could address this in the face of uncertainty is to flip the question and ask how different EV end of life pathways would need to be in order to change the conclusion of the paper.

> **Response:** Thank you for suggesting we explore how secondary markets could affect our conclusions. The pathway needed to *"change the conclusion[s]"* is likely physically implausible. This would require the small amount of exported and second life application BEVs to accumulate enough emissions to outweigh the overall lifecycle emissions of the whole BEV fleet (including those not exported or with second life).
>
> A brief thought experiment shows this is likely physically implausible: assuming the mean BEV accumulates 20t CO_2e before it is exported or reaches second life applications, would need to accumulate at least a further 15t CO_2e to reach HEVs. Normalising this on a per kilometre basis, or factoring in that HEVs accrue more emissions in second hand markets, would increase this already-high requirement further. To flip the conclusions of the paper, this exported and second life share of BEVs would need to offset the remaining 85% of vehicles that were not exported or have second life applications, and so would need to accumulate six times the emissions: a further 90t CO_2e. If this were supplied entirely by coal electricity generation alone, it would require approximately another 500,000 km of vehicle mileage. Therefore, have amended the text in the paragraph covering exports and battery second life to include:
>
> *"Furthermore, it is unplausible that vehicle exports and battery second life could change our overall conclusions: that BEVs provide lower lifecycle emissions per km than HEVs, petrol, and diesel cars."*

2) Grid intensity: The use of attributional LCA and ISO 14040/14044 is not suitable for assessing the effect of changes, including the effect of improving EV mileage and lifetime identified in the paper's title. This is what consequential LCA is for.

> **Response:** See above comments on consequential LCA.

3) Transformer predictions: The SI compares actual and predicted annual vehicle mileage across the fleet after the BEVs and HEVs have been reclassified as petrol or diesel vehicles. There are a few issues: (1) the predictions are systematically biased (e.g.: in Figure S3c the colored area is consistently above the 1:1 line for low mileage vehicles and consistently below the 1:1 line for high mileage vehicles); (2) it is not clear exactly what test is performed, but it looks like these are a within-sample fit statistics when what is needed is out-of-sample prediction error, such as cross validation; and (3) for the application, it is not enough that the model can predict the bulk fleet after the BEVs and HEVs have been reclassified as petrol and diesel vehicles; the claims are about future BEV performance specifically, so validation needs to be performed on predicting BEV mileage, lifetime, scrap rates, etc.

> **Response:** Thank you for highlighting the need to improve the clarity of model training, validation, and performance. We address each of these points in turn below.
>
> 1) We acknowledge there is systematic bias in our model. This pattern, overestimation at low values and underestimation at high values, is a well-documented inherent property of ML regression models arising from the bias-variance trade-off, common across all ML architectures. We have revised the text to acknowledge this explicitly and to explain why the approximate symmetry of this bias in our model means it does not materially affect our fleet-level estimates. The revised text in the methods now reads:
>
> *"The mileage predictions exhibit some systematic bias, overestimation for low-mileage vehicles and underestimation for high-mileage vehicles (Figure S3c). This pattern of regression toward the mean is a well-documented and inherent property of machine learning regression models arising from the bias-variance trade-off and has been shown to be common across diverse ML architectures rather than specific to any particular model[6]. As this bias is approximately symmetric about the mean, the overestimation and underestimation largely cancel at the fleet-aggregate level, and the aggregate errors are modest relative to observed variability and do not materially affect fleet-level mileage and lifetime and the upstream emissions estimates. "*
>
> 2) For model training and validation, we use standard practice of using a split test where the training data is split into 80% for training and the remaining unseen out-of-sample data is used for validation metrics on this unseen data. This is now more clearly explained in the text in the methods:
>
> *"The model was validated using a split test, where 80% of the sequences were used to train the model and the remaining unseen out-of-sample data was used for model validation. The performance of the mileage regression prediction is illustrated in Figure S3, and the performance metrics of the mileage regression and survival probability classification are given in Table S6. This validation reveals the model performs robustly on unseen data. "*
>
> 3) Thank you for the suggestion to extend validation to HEVs and BEVS. We have added additional validation of our model specifically for BEVs and HEVs. This validation consists of a new leave-last-value-out test which demonstrates that model is able to capture BEV and HEV mileage and survival. We have added a new paragraph in the methods providing this validation.
>
> *"To validate the model's performance on BEVs and HEVs we conducted a leave-last-value-out test. For each HEV and BEV sequence for vehicles aged 4 and over, the final real-world*

> *MOT test record was withheld, and the model was used to predict both annual mileage (regression) and survival probability (classification) for that held-out test using only the preceding records. Prediction performance on these withheld BEV and HEV tests was comparable to the results obtained on petrol and diesel vehicles and are given in Table S7. This evaluation on the most recent tests was appropriate because mileage and survival trends for BEVs and HEVs are converging toward those of petrol and diesel vehicles in recent years (Figures 2 and 3). These results confirm that the model is suitable for projecting BEV and HEV mileage, survival, and emissions."*
>
> We have provided the metrics in the Supplementary Information Table S7, which shows these validation metrics for BEVs and HEVs.

4) Causality: As noted above, the paper's framing is causal, despite the added caveats. It asks a causal question: how increases to EV mileage and lifetimes change lifecycle emissions benefits.

> **Response:** See above comments on consequential LCA and causality.

5) Batteries: Changes in batteries between BEVs sold in 2012 and 2023 include far more than just range. Battery chemistry, coatings, microstructure, thermal management, controls, cell format, production improvements, and other factors have all changed over this period, and all of these things affect battery life. For LFP cells, summarizing use via equivalent full cycles is reasonable, but for NMC degradation depends too heavily on other factors, such as depth of discharge, c-rate, temperature, etc. to be summarized this way, and larger packs will differ in depth of discharge, heat generation, etc. relative to smaller packs over the same drive cycle.

> **Response:** Thank you for providing further insight into battery technology. We have revised this paragraph to include further discussion on the subject that you raise. We add a specific recommendation for future work that could incorporate more factors on the underlying battery technology for each BEV in our data, enabling better understating for how battery technology impacts vehicle longevity that you raise.
>
> However, we wish to reiterate that this paragraph is there to provide some context: when delivering the same energy throughput, a smaller battery will by cycled more frequently than a large battery, and that will translate to higher degradation. Other factors will influence this, but we are simply using some numbers to make this point, and clearly label this "a simple thought experiment". So, piling more detail in will not further than aim.
>
> We have edited the paragraph to:
>
> *"Our results suggest that BEVs registered in 2022-2024 may equal the lifetime mileage of petrol cars if current trends continue. Both usage and lifetime trends might partly reflect increasing battery capacity. Larger batteries enable longer journeys, making newer BEVs more versatile and thus used more. Battery degradation reduces useable capacity over time, further reducing range. As a simple thought experiment, acknowledging that real-world degradation varies substantially by chemistry and operating conditions, if capacity falls by 1.8%-2.3% per year[7–9], BEVs from 2012 would have lost up to 25% by 2023, exceeding the*

> *typical 20% end-of-life threshold*[10,11]*. Combining the 2012 cohort's mean battery size (22 kWh) and energy consumption (5.9 km per kWh) gives an average range reduction from 130 km to 98 km, which might help explain why 45% of this cohort had exited the fleet by 2024. Smaller batteries are also cycled more intensively per kilometre driven, implying faster degradation*[12]*. The 2012 cohort had accumulated a mean of 700 equivalent full cycles by 2024, and by age 5 they reached 350 cycles, with only 74% remaining. In contrast, the 2019 cohort (with 60 kWh capacity and 350 km range) had accumulated only 190 cycles by the same age, and 93% remained in 2024. These differences reflect not only pack size but also technological advances between 2012 and 2021 which affect battery longevity. Future research could improve our vehicle specifications to provide the battery technology for each BEV enabling greater understanding for how battery technology effects overall longevity. "*

Overall, I think there is some very interesting data and analysis in this study, and I think it could do well in a transportation journal with some revision to address the comments above. I just think that the big-picture claims that can be supported by this analysis are too caveated and limited to be a good match for a broad-interest journal.

> **Response:** Thank you for taking the time to review our study. We believe our work has been improved through your comments.

Response to Reviewer #3

The information presented in this paper is very important and relevant as it closes a research gap. It provides information on annual mileage and survival rates of passenger cars separately by powertrain type. Especially the information for battery electric vehicles (BEVs) presents new evidence showing that older BEVs have a much shorter lifetime and lower annual mileage than internal combustion engine vehicles (ICEVs), whereas BEVs registered today are used similar to ICEVs, having similar survival rates and lifetime mileages - a very important finding to inform life-cycle and total cost of ownership analyses.
The work is presented in a transparent and comprehensible manner. It is great that the author makes both the source data and code publicly available. This enables conducting the analysis for other countries or markets.

> **Response:** Thank you for providing your comments and your kind words of appreciation for our study. We believe your input has improved this study.

Thank you for addressing my comments thoroughly. After the 2nd review, I have only three comments remaining:
* The small dots in Figure 1a are almost not discernible and the ones in yellow are almost not visible. I suggest: using larger dots; grouping "United Kingdom" with the other countries/regions and not "Global"; potentially using a different symbol for global which would allow using a different scaling for the countries/regions than for the global symbol.

> **Response:** Thank you for highlighting the issue with figure 1a. We have experimented with various dot scales up to 4x that used in the existing figure, but none were sufficient to make the smaller dots of Oceania and South America clearly visible. Therefore, we have decided to remove these from the figure. Following your feedback, we have also removed the UK from this figure, including the UK within the values for Europe. These changes are included in the revised Figure 1.

* On page 14, the high mileage of HEVs is discussed. In some countries, Toyota HEVs are often used for ride-hailing services (Taxis, UBER etc). This could explain the high annual mileages.

> **Response:** Thank you for highlighting the usage of HEVs as private hire vehicles and taxis. We found it was possible to test this idea, because private hire vehicles (PHVs) and taxis are typically required to have an MOT test every half a year, depending on the local authority. Thus, we can filter the data for vehicles that have between 0.4 and 0.6 years to provide an approximate population of vehicles used as PHVs and taxis. This has allowed us to the existing paragraph covering HEV mileage:
>
> *"Annual mileage for HEVs was similar to diesel vehicles in earlier model years but is declining in more recent years. Both diesel and HEVs have lower fuel consumption than petrol vehicles, making them more economical for high mileage users, including commercial applications such as Private Hire Vehicles (PHVs) and taxis. The high mileage observed for HEVs might therefore be explained by a larger proportion of these vehicles being used in PHV and taxi services. Based on the MOT data, we estimate that in 2015, 13% of the HEV*

> *fleet aged 3–8 years were PHVs or taxis, far exceeding the 2-3% share for petrol and diesel vehicles. In 2015, the annual mileage of HEV PHVs and taxis were approximately twice the mean for diesel and HEVs (39,500 cf. 16,500 and 19,200 respectively), and three times the average for petrol (11,200). By 2024, the share of HEVs used as PHVs or taxis had declined to 5%, although these vehicles continued to record similarly increased annual mileage as in 2015.*
>
> *Policy incentives and consumer trends could have also influenced vehicle usage patterns. For example, exemptions from London's congestion charge for HEVs and BEVs[1] potentially encouraged higher mileage among these vehicles. In addition, the rising share of micro-HEVs and mild-HEVs, which provide smaller fuel-economy benefits than full hybrids[2,3], could have contributed to the decline in mean annual mileage for the overall HEV category (which includes micro, mild and full hybrids). Future work could investigate these influences, but the latter would require more granular data on vehicle classification."*
>
> We have added to the methods how we filtered the MOT data for these vehicles:
>
> *"Most vehicles operating as a private hire vehicle or taxi require an MOT test frequency of 6 months[4]. To produce an approximation of the population of private hire vehicles and taxis we filter the MOT data for vehicles that have had a test within 0.4 to 0.6 years. This filtering step will include a portion of vehicles that coincidentally have this time between MOT tests. Some local authorities have differing rules regarding MOT test frequency and so we cannot identify all taxis and private hire vehicles with this method. "*

Vehicles with high annual mileages often show superior durabilty as they are operated much less of their lifetime in cold start mode (cold coolant/cold oil) and are serviced regularly. Therefore, the mentioned longevity might at least partly owed the vehicle usage and not only a better build and design quality. If this is the case in the UK, it could be mentioned.

> **Response:** Thank you for highlighting this aspect of vehicle longevity. We have rearranged the two paragraphs on HEVs in this section, so we now cover annual mileage first and then lifetime to allow us to include this point where the higher usage rate of HEVs may influence longevity:
>
> *"The higher mileage of HEVs may also contribute to better longevity since vehicles will spend a larger proportion of their in hot-engine conditions."*

* The last sentence in the same paragraph refers to micro- and mild-HEVs. It is difficult to understand that this sentence provides the reason for the declining annual mileage of newer HEVs. Consider rephrasing. And the sentence gives the impression that consumers opt intentionally for a micro- or mild-hybrid (MHEVs) instead of a full-hybrid. However, MHEVs replace more and more conventional combustion engine vehicles (ICEV) and consumers might choose an MHEV without being aware of the hybridization, assuming they buy an ICEV.

> **Response**: Thank you for highlighting how the text is poorly phrased in this section. We have amended the end of the paragraph to now read:

> *"In addition, the rising share of micro-HEVs and mild-HEVs, which provide smaller fuel-economy benefits than full hybrids[2,3], could have contributed to the decline in mean annual mileage for the overall HEV category (which includes micro, mild and full hybrids). Future work could investigate these influences, but the latter would require more granular data on vehicle classification."*

References

1. Knobloch, F. *et al.* Net emission reductions from electric cars and heat pumps in 59 world regions over time. *Nat. Sustain.* 3, 437–447 (2020).
2. Santero, N. *et al.* Electrifying light vehicles in the United States shows emission reduction potential for all vehicle types and powertrains. *Commun. Sustain.* 1, 23 (2026).
3. Šimaitis, J. *et al.* Battery electric vehicles show the lowest carbon footprints among passenger cars across 1.5–3.0 °C energy decarbonisation pathways. *Commun. Earth Environ.* 6, 476 (2025).
4. Horesh, N., Trinko, D. A. & Quinn, J. C. Comparing costs and climate impacts of various electric vehicle charging systems across the United States. *Nat. Commun.* 15, 4680 (2024).
5. Nguyen-Tien, V., Zhang, C., Strobl, E. & Elliott, R. J. R. The closing longevity gap between battery electric vehicles and internal combustion vehicles in Great Britain. *Nat. Energy* 1–11 (2025) doi:10.1038/s41560-024-01698-1.

Response to Reviewers

We thank the reviewers for their constructive comments that have helped to improve this manuscript. We agree with and have actioned all comments from the reviewers, including a thorough rewording of the text to remove any ambiguity regarding the attributional lifecycle analysis and how the results should be interpreted.

To summarise our main changes to the manuscript and supporting files:

- Rewording of the Abstract, Introduction, Results, Discussion, and Methods to avoid ambiguity relating to causality and to be explicit in all claims that our study provides attributional lifecycle emissions.
- Revised Supplementary Information section titles and captions.

Below, we respond to each reviewer's comment individually, and quote changes to the manuscript in *italics*.

Response to Reviewer #1

The authors thoroughly addressed my comments providing additional insights and analyses. I have no further comments.

> Thank you for your previous helpful comments on our work.

Response to Reviewer #2

I appreciate the efforts that the authors have put in to making their case, but in my view the arguments are not persuasive on the central question of causality.

> **Response:** Thank you for raising the central concern about causality. We revised the manuscript to make the attributional scope explicit and to avoid presenting attributional lifecycle estimates as causal estimates of replacement decisions.
>
> We have revised the Abstract, Introduction, Results (lifecycle greenhouse gas emissions), Discussion, and Methods.
>
> For example:
>
> *"Battery electric vehicles (BEVs) are typically estimated to have lower attributional lifecycle greenhouse gas emissions than conventional internal combustion engine vehicles."*
>
> *"We modelled attributional lifecycle emissions by combining manufacturing emissions, based on vehicle mass and battery capacity, with use-phase emissions derived from MOT mileage data, and the GHG intensity of British electricity production."*
>
> *"A central uncertainty in vehicle lifecycle assessment is whether real-world electric vehicles are driven as intensively and for as many years as the conventional vehicles they are compared to in lifecycle analyses."*

The central focus of the study is how new data on UK vehicle travel patterns and survival rates affects attributional life cycle estimates that have previously relied on assumptions for these factors. This is a fine thing to study, but it does not answer the larger questions claimed.

> **Response:** Thank you for clarifying this distinction. We revised the manuscript so the contribution is framed as an empirical improvement to attributional lifecycle estimates, rather than as an answer to broader consequential questions.
>
> We have revised the Abstract, Introduction, and Discussion.
>
> For example:
>
> *"Here we use data for every electric vehicle in Britain to assess mileage and lifetime compared to conventional and hybrid vehicles, and the implications for attributional lifecycle greenhouse gas emissions."*
>
> *"We combine data on vehicle mileage and lifetime with vehicle attributes, such as fuel economy, battery size, and vehicle mass, to calculate attributional emissions of vehicles over their lifetime."*

The revised title is improved in that it no longer makes a causal claim about the effects of improved mileage and lifetime on emissions. But the first sentence of the abstract returns to causal language: "Battery electric vehicles (BEVs) are predicted to reduce lifecycle greenhouse gas emissions compared to conventional vehicles."The claim that BEVs "reduce" lifecycle greenhouse gas emissions compared to conventional vehicles is causal: implying that the action of replacing a conventional vehicle with a BEV will result in reduced net GHG emissions. The use of the verb "reduce" makes this a consequential claim, not an attributional one, and answering it requires assessing how emissions change (e.g.: "reduce") when conventional vehicles are replaced with BEVs. A corrected attributional claim would read something more like the following: "Attributional lifecycle assessments of conventional and electric vehicles typically report lower GHG emissions estimates for electric vehicles than for gasoline vehicles." These attributional assessments are notably *not* estimates of how much emissions are "reduced" when conventional vehicles are replaced with BEVs. Rather, they represent a particular accounting framework filled with a combination of empirical data and modeling choices, such as where to draw a geographic boundary in an interconnected power grid to determine which emissions to assign to EV charging load in a particular region. Attributional LCA involves arbitrary modeling choices about how to assign process emissions to coproducts, and the power grid has the largest collection of coproducts of any system, so the distinction between attributional estimates and causal claims is especially sharp.

> **Response:** Thank you for identifying the causal wording in the opening sentence of the abstract and the additional clarification for how we should frame our work in other sections. We revised this sentence to use attributional lifecycle language while retaining a direct abstract opening.
>
> We have revised the Abstract to read:

> *"Battery electric vehicles (BEVs) are typically estimated to have lower attributional lifecycle greenhouse gas emissions than conventional internal combustion engine vehicles."*

Similarly, the last sentence of the abstract claims that BEVs "may produce" 99 g CO2 per km over their lifetime and that this confirms "their climate change mitigation potential." Again, this claim is causal: implying that owning and operating an EV may "produce" a particular amount of emissions (relative to not owning an operating an EV) and that these attributional estimates are relevant for estimating the "mitigation potential" of switching from conventional vehicles to BEVs. Estimating mitigation potential requires estimating how much emissions mitigation is possible (e.g.: by replacing conventional vehicles with BEVs). Attributional LCA does not provide estimates of this because it does not attempt to estimate how emissions would change if conventional vehicles were replaced with BEVs.

> **Response:** Thank you for pointing out this issue in the final abstract sentence. We retained the numerical estimate but removed the claim that it confirms mitigation potential, and we now state it as a projected attributional lifecycle-emissions estimate under the same accounting framework.
>
> We have revised these statements to read:
>
> *"Training a transformer neural network on this national data, we estimate that current BEVs have projected attributional lifecycle emissions of 99 g CO_{2e} per kilometre over their lifetime, less than half of the 244–246 g for conventional vehicles and 195 g for hybrid vehicles, when accounting for their real-world usage."*
>
> *"We find that on average, current BEVs have projected attributional lifecycle emissions of 99 g CO_{2e} per kilometre, 60% lower than previous BEVs which have already exited the fleet."*

Overall, the authors conduct an attributional assessment, but they use language throughout suggesting that they incorrectly interpret those attributional estimates as though they are causal and consequential. My view is that cleaning up this language to avoid any causal or consequential claims would make the paper accurate, and the contribution would be valuable to other life cycle modelers who conduct attributional LCA and would like to understand how new data on travel patterns and survival might affect their estimates. But because the analysis does not answer the causal questions that it currently claims, I do not think it is well suited for a broader audience. It would be better situated in an LCA journal because its primary audience is other LCA researchers, not researchers outside the field or policymakers wanting to understand the emissions implications of transitioning to BEVs.

> **Response:** Thank you for this broader framing comment. We revised the manuscript throughout to avoid treating attributional lifecycle estimates as causal or consequential estimates. However, we respectfully disagree with your view that a carefully scoped attributional analysis is only of interest to LCA specialists. Lifecycle analyses are regularly used in broad energy, transport, and resource debates when they address empirically important uncertainties; attributional LCA examples include work published in broad Nature Portfolio venues (for example, Knobloch et al., 2020; Machala et al., 2025; Morfeldt & Johansson, 2022) and lifecycle assessment is used by the International Resource Panel

to identify global resource and product priorities. Our revisions therefore narrow the manuscript's claims while preserving the broader relevance of the empirical contribution.

We have made revisions throughout the manuscript, for example:

"Battery electric vehicles (BEVs) are typically estimated to have lower attributional lifecycle greenhouse gas emissions than conventional internal combustion engine vehicles. These estimates rely upon lower usage emissions outweighing higher manufacturing emissions, but limited data on how intensively electric vehicles are used, and how long they operate for, casts uncertainty over comparisons."

"Together, these show that newer cohorts of conventional and hybrid vehicles have lower attributed cumulative GHG emissions over time (comparing blue to red lines in Figure 4a), which is in part due to improved fuel economy (comparing blue to red dots in Figure 4b), and partly due to reduced utilisation."

"This work shows that current-generation battery electric vehicles produce less than half the attributional lifecycle greenhouse gas emissions of conventional vehicles, based on real-world use by British drivers."

References:

Machala, M. L. *et al.* Life cycle comparison of industrial-scale lithium-ion battery recycling and mining supply chains. *Nat. Commun.* **16**, 988 (2025).

Knobloch, F. *et al.* Net emission reductions from electric cars and heat pumps in 59 world regions over time. *Nat. Sustain.* **3**, 437–447 (2020).

Morfeldt, J. & Johansson, D. J. A. Impacts of shared mobility on vehicle lifetimes and on the carbon footprint of electric vehicles. *Nat. Commun.* **13**, 6400 (2022).

My recommendation remains that this paper should strictly avoid causal claims about how BEVs might "reduce" emissions relative to conventional vehicles or offer "mitigation potential", since attributional LCA does not estimate causal effects like these. Rather, the paper estimates how new data about lifetime and travel patterns affect attributional LCA estimates of BEVs and conventional vehicles. The paper will have value in an LCA journal, but without legitimate causal claims relevant to emissions reductions and climate change mitigation potential, I am skeptical of its value to a broader audience such as that targeted by Nature Communications.

Response: Thank you for restating this concern. We revised the manuscript to avoid causal claims about emissions reductions or mitigation potential and to state the analysis as an attributional lifecycle assessment using observed mileage and survival data. We do not agree that this makes the paper relevant only to an LCA-specialist audience: the central empirical question is whether observed use and survival of BEVs support a key assumption used in lifecycle comparisons, which is relevant to transport, energy, climate, and data-driven sustainability research beyond specialist LCA methodology.

We have made revisions throughout the manuscript, for example:

> *"Attributional lifecycle emissions comparisons rely on four factors: embodied manufacturing emissions, use-phase emissions from electricity generation supplying the vehicles, how much vehicles are driven, and how long vehicles last."*
>
> *"BEVs therefore had 9-20% lower attributed emissions per kilometre driven than petrol and diesel vehicles over their lifespan, despite the lower mileage and shorter lifetime of these older BEVs, and the higher carbon intensity of British electricity in the 2010s."*

Response to Reviewer #3

Thank you for addressing my comments. As mentioned before, this publication will close a knowledge gap and will be helpful for future research and policy work. I therefore recommend the publication of this report.

> Thank for your kind words of appreciation for our study.

I would also like to incourage the authors to regularly publish updated numbers on lifetime mileage and survival curves, considering the fast paced developments observed for electric vehicles.

> **Response:** Thank you for this helpful suggestion. We are happy to update the public repository as new MOT data releases become available, and we added a concise statement to the Code Availability section:
>
> *"Code is available from: https://github.com/DanielMehlig/ev-mileage-and-lifetime. We intend to update the public repository with revised mileage and survival estimates as new MOT data releases become available."*

My remaining comments only address typos and reference errors:

* In line 387, there is a word missing after "their"

> **Response:** Thank you for finding this error. We have corrected this sentence to be:
>
> **"***The higher mileage of HEVs may also contribute to better longevity since vehicles will spend a larger proportion of their operation in hot-engine conditions."*

* The references to the supplementary information need to be checked. E.g. the main document refers to Table 6 in the supplement, but tables are labeled as e.g. Table S6. Furthermore, additional information was introduced in the supplement, so table numbering changed. This also led to two tables being labeled as S7.

> **Response:** Thank you for highlighting this error. We have corrected these cross-references, and the captions and headings used in the Supplementary Information. The Supplementary Information and main text are now correct and use the appropriate conventions for this journal.

Response to Reviewers

We thank the reviewers for their constructive comments that have helped to improve this manuscript. We agree with the final comment from Reviewer 2 and have added the recommended passage to the text.

Below, we respond to each reviewer's comment individually, and quote changes to the manuscript in *italics*.

Response to Reviewer #2

The authors have addressed my comments by rewording text across the manuscript to appropriately contextualize the results within attributional LCA and to avoid making causal claims. They also provided evidence of other studies in Nature Communications and Nature Sustainability that rely on attributional LCA in order to argue for broad relevance despite the absence of causal claims. These are all reasonable.

> **Response:** Thank you for your comments on our work.

My remaining request is that the authors include a sentence when discussing limitations of the study indicating that future work on causal and consequential analysis could leverage these results to help estimate emissions implications of corporate, consumer or policy actions, such as encouraging increased BEV adoption or influencing vehicle scrappage patterns.

> **Response:** Thank you for this suggestion. We have added this passage to the closing paragraph of the discussion:
>
> *"Finally, our lifecycle emission results are attributional, providing an account of current fleet emissions rather than the effect of specific actions. Future work could use causal or consequential methods to estimate how actions such as encouraging BEV adoption or altering scrappage patterns would affect emissions."*
